# Mutations in the *Caenorhabditis elegans* orthologs of human genes required for mitochondrial tRNA modification cause similar electron transport chain defects but different nuclear responses

**DOI:** 10.1371/journal.pgen.1006921

**Published:** 2017-07-21

**Authors:** Carmen Navarro-González, Ismaïl Moukadiri, Magda Villarroya, Ernesto López-Pascual, Simon Tuck, M.-Eugenia Armengod

**Affiliations:** 1 Modificación del RNA y Enfermedades Mitocondriales, Centro de Investigación Príncipe Felipe, Valencia, Spain; 2 Umeå Center for Molecular Medicine, Umeå University, Umeå, Sweden; 3 Centro de Investigación Biomédica en Red de Enfermedades Raras (CIBERER), node U721, Valencia, Spain; University of Cologne, GERMANY

## Abstract

Several oxidative phosphorylation (OXPHOS) diseases are caused by defects in the post-transcriptional modification of mitochondrial tRNAs (mt-tRNAs). Mutations in *MTO1* or *GTPBP3* impair the modification of the wobble uridine at position 5 of the pyrimidine ring and cause heart failure. Mutations in *TRMU* affect modification at position 2 and cause liver disease. Presently, the molecular basis of the diseases and why mutations in the different genes lead to such different clinical symptoms is poorly understood. Here we use *Caenorhabditis elegans* as a model organism to investigate how defects in the *TRMU*, *GTPBP3* and *MTO1* orthologues (designated as *mttu-1*, *mtcu-1*, and *mtcu-2*, respectively) exert their effects. We found that whereas the inactivation of each *C*. *elegans* gene is associated with a mild OXPHOS dysfunction, mutations in *mtcu-1* or *mtcu-2* cause changes in the expression of metabolic and mitochondrial stress response genes that are quite different from those caused by *mttu-1* mutations. Our data suggest that retrograde signaling promotes defect-specific metabolic reprogramming, which is able to rescue the OXPHOS dysfunction in the single mutants by stimulating the oxidative tricarboxylic acid cycle flux through complex II. This adaptive response, however, appears to be associated with a biological cost since the single mutant worms exhibit thermosensitivity and decreased fertility and, in the case of *mttu-1*, longer reproductive cycle. Notably, *mttu-1* worms also exhibit increased lifespan. We further show that *mtcu-1*; *mttu-1* and *mtcu-2; mttu-1* double mutants display severe growth defects and sterility. The animal models presented here support the idea that the pathological states in humans may initially develop not as a direct consequence of a bioenergetic defect, but from the cell’s maladaptive response to the hypomodification status of mt-tRNAs. Our work highlights the important association of the defect-specific metabolic rewiring with the pathological phenotype, which must be taken into consideration in exploring specific therapeutic interventions.

## Author summary

Post-transcriptional modification of tRNAs is a universal process, thought to be essential for optimizing the functions of tRNAs. In humans, defects in the modification at position 2 (performed by protein TRMU) and 5 (carried out by proteins GTPBP3 and MTO1) of the uridine located at the wobble position of mitochondrial tRNAs (mt-tRNAs) cause oxidative phosphorylation (OXPHOS) dysfunction, and lead to liver and heart failure, respectively. However, the underlying mechanisms leading to pathogenesis are not well-known, and hence there is no molecular explanation for the different clinical phenotypes. We use *Caenorhabditis elegans* to compare in the same animal model and genetic background the effects of inactivating the TRMU, GTPBP3 and MTO1 orthologues on the phenotype and gene expression pattern of nuclear and mitochondrial DNA. Our data show that *C*. *elegans* responds to mt-tRNA hypomodification by changing in a defect-specific manner the expression of nuclear and mitochondrial genes, which leads, in all single mutants, to a rescue of the OXPHOS dysfunction that is associated with a biological cost. Our work suggests that pathology may develop as a consequence of the cell’s maladaptive response to the hypomodification status of mt-tRNAs.

## Introduction

Mitochondria are essential bioenergetic and biosynthetic eukaryotic organelles. Via oxidative phosphorylation (OXPHOS), they produce most of the cellular ATP; via the tricarboxylic acid cycle, they generate intermediate metabolites and reducing equivalents (NADH and FADH_2_). The coupling between mitochondrial activity and cell physiology depends on anterograde and retrograde signaling pathways. The latter one influences nuclear gene expression in response to the mitochondrial functional state. Retrograde signaling changes in response to signaling molecules released by mitochondria such as Ca^2+^ and reactive oxygen species (ROS). It is also affected by AMP/ATP and NAD^+^/NADH ratios (which are dependent upon mitochondrial function), and by peptides produced by fragmentation of mitochondrial proteins [1–4]. In these ways, mitochondria play critical roles in cell cycle progression, differentiation, development, immune responses, and apoptotic cell death [5].

The OXPHOS system consists of five complexes (CI-CV) embedded in the mitochondrial inner membrane, and two mobile electron shuttles, Coenzyme Q (CoQ) and cytochrome *c*. Complexes CI to CIV (respiratory complexes) oxidize the electron carriers NADH and FADH_2_ in a process that is coupled to the pumping of protons by CI, CIII and CIV into the intermembrane space. The resulting proton gradient is used by CV to synthesize ATP. NADH reducing equivalents are funneled into the OXPHOS system through CI, whereas FADH_2_ reducing equivalents are incorporated through CII, or diverse electron transport flavoproteins (ETFs), without creating a transmembrane proton gradient [6].

In human mitochondria, 13 key OXPHOS subunits are encoded by the mitochondrial DNA (mtDNA), which also encodes the 22 tRNAs and 2 rRNAs responsible for the intra-mitochondrial protein synthesis. The remaining OXPHOS subunits and all proteins required for proper functioning of the mitochondrial translational system, such as mitoribosomal proteins, translation factors, aminoacyl tRNA synthetases, and RNA modifying proteins, are encoded by the nuclear DNA (nDNA). Mutations in nuclear or mitochondrial genes that are required for mitochondrial translation cause diseases that are associated with OXPHOS dysfunction and a variety of tissue-specific phenotypes [7]. In particular, mutations in *TRMU* (MIM*610230), *GTPBP3* (MIM*608536), and *MTO1* (MIM*614667), which affect the post-transcriptional modification of the uridine located at the wobble position (U_34_) of certain mt-tRNAs, have been found to be associated with liver (*TRMU*) and heart failure (*GTPBP3* and *MTO1*) [8–12]. A TRMU mutation has also been shown to worsen the phenotype caused by a deafness-associated mitochondrial 12S rRNA mutation [13, 14].

**Fig 1:**
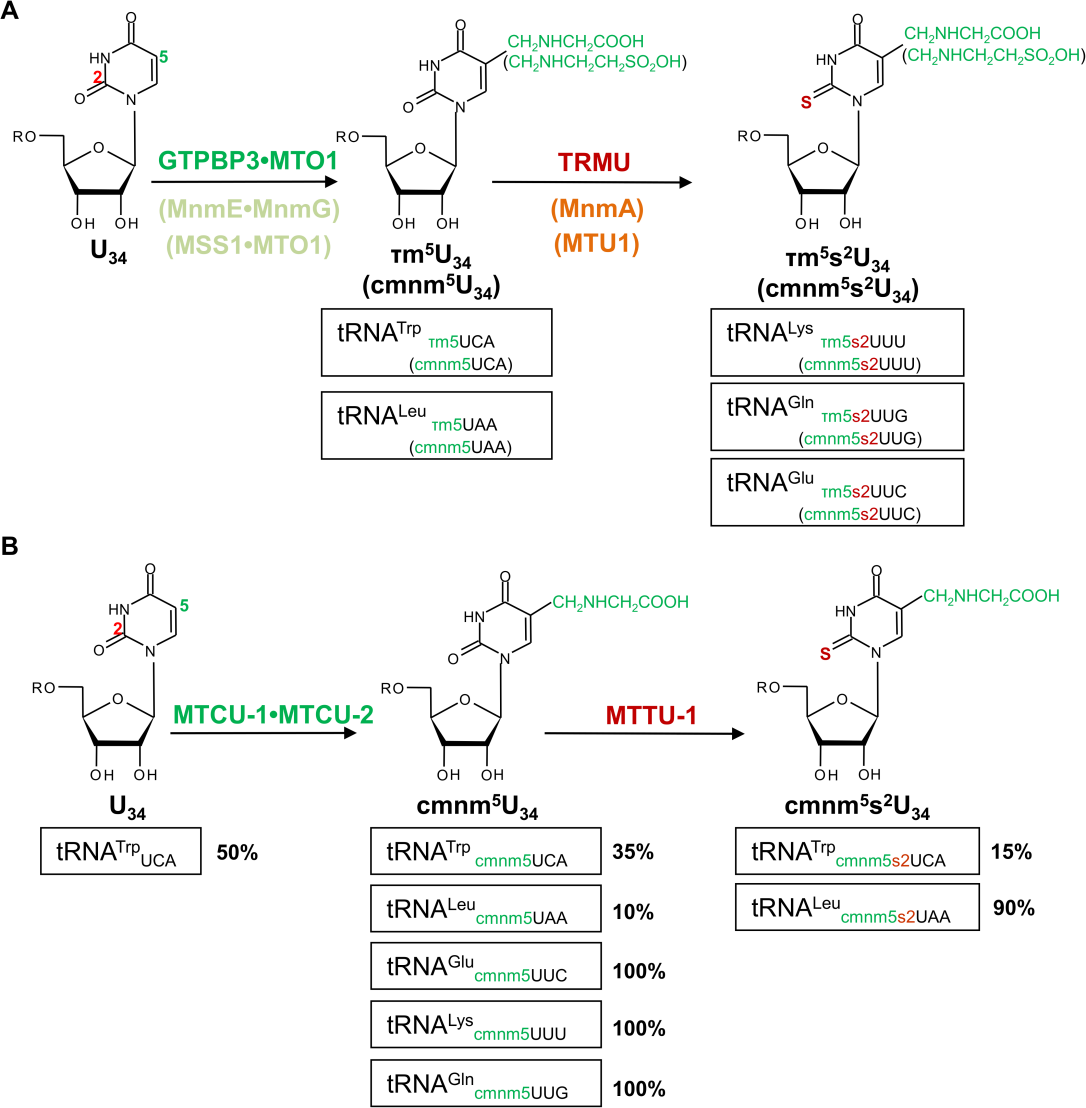
Modification of the wobble uridine (U_34_) in mitochondrial and bacterial tRNAs. Schema of the U_34_ modification pathways in human and yeast mt-tRNAs and *Escherichia coli* tRNAs **(A)** and *A*. *suum* mt-tRNAs **(B)**. In A, proteins GTPBP3, MTO1, and TRMU (also named MTU1) from humans, and MSS1, MTO1, and MTU1, from yeast, are orthologous of the bacterial MnmE, MnmG and MnmA proteins, respectively. Taurine (humans) and glycine (*E*. *coli* and yeast) are used to introduce the τm and cmnm groups into position 5 of U_34_. In B, MTCU-1, MTCU-2 and MTTU-1 are the nematode orthologues of GTPBP3, MTO1, and TRMU, respectively, and their roles are inferred from studies of the *E*. *coli* and yeast proteins. The fractions of *A*. *suum* modified mt-tRNAs, according to the study by Sakurai *et al*. [28], are indicated below the schema. Note that mt-tRNA^Glu^, mt-tRNA^Lys^ and mt-tRNA^Gln^ are fully modified at position 5 but lack thiolation at position 2, whereas most ${\text{mt-tRNA}}_{\text{UAA}}^{\text{Leu}}$ (~90%) is modified at both positions (2 and 5), and about 50% of mt-tRNA^Trp^ molecules do not contain any modification at the U_34_.

From studies of their bacterial or yeast orthologues, GTPBP3 and MTO1 are predicted to catalyze the addition of the taurinomethyl group at position 5 of the U_34_ in human mt-tRNAs decoding for Lys, Glu, Gln, Leu^(UUR)^, and Trp. TRMU (also named MTU1), on the other hand, thiolates position 2 of U_34_ in a subgroup of these mt-tRNAs (mt-tRNA^Lys^, mt-tRNA^Glu^, and mt-tRNA^Gln^) (Fig 1A) [8, 15–18]. It should be pointed out, however, that, since limited material is available, precise quantification of the level of these modifications in mt-tRNAs from patient cells has not been conducted to date. In any case, defects in GTPBP3, or MTO1, or TRMU compromises mitochondrial translation to some extent and lead to OXPHOS dysfunction, proteostasis stress, and activation of retrograde signaling pathways [8, 11, 18–21]. Presently, however, it is very unclear how *TRMU* mutations lead to liver disease while those in *GTPBP3* and *MTO1* lead to heart failure [8–11]. An additional problem in the study of these diseases is that a global impairment of mitochondrial translation is not always apparent in some types of patient cells or in disease cell models [9, 11, 12, 15, 18]. Moreover, it has been recently shown that MTO1-deficiency induces an imbalance in AKT, mTOR and AMPK retrograde signaling in all tissues of a mouse model independent of the OXPHOS defect [20]. Therefore, the correlation between the mt-tRNA hypomodification level, mitochondrial translation impairment, OXPHOS deficiencies and clinical symptoms remains to be clarified. In this context, we hypothesize that retrograde signaling triggered by the inactivation of *TRMU*, *GTPBP3* or *MTO1* is different, which results in different tissue-dependent nuclear responses and, consequently, in different phenotypes at the organismal level. To test this hypothesis it will be crucial to use a model organism that minimizes genotypic differences among individuals carrying inactivating mutations in *TRMU*, *GTPBP3* or *MTO1*.

The small free-living nematode *Caenorhabditis elegans* has been demonstrated to be a powerful model organism for the study both of OXPHOS defects and of the consequences of the mitochondrial dysfunction [22–27]. No reports exist presently, however, describing either the types of modification found on mitochondrial tRNAs or the *C*. *elegans* orthologues of *TRMU*, *GTPBP3* or *MTO1*. In this organism, twelve proteins, all components of the complexes making up the OXPHOS system, are mitochondrially encoded. The *C*. *elegans* mitochondrial genome also encodes two ribosomal RNAs and 22 tRNAs and, although lacking the ATP8 gene, is very similar in its coding capacity to the human mitochondrial genome.

While U_34_ modifications of *C*. *elegans* mt-tRNAs have not previously been reported, such modifications have been studied in the much larger nematode *Ascaris suum*, a parasite of pigs [28, 29]. In this organism, the tRNAs thiolated at position 2 are different from those in yeast and humans [16]. In *A*. *suum*, ${\text{mt-tRNA}}_{\text{UAA}}^{\text{Leu}}$ and mt-tRNA^Trp^ are thiolated but not those mt-tRNAs decoding Lys, Glu and Gln, which are thiolated in yeast and humans (Fig 1). In contrast, these five mt-tRNA species of *A*. *suum* carry at position 5 of U_34_ the modification expected to be introduced by the GTPBP3 and MTO1 orthologues (Fig 1B).

In this work, we develop *C*. *elegans* as a model to study mt-tRNA modification and the functions at both cellular and organismal levels of human disease gene orthologues involved in this process. We demonstrate that mutations affecting thiolation or carboxymethylaminomethylation of wobble uridines in mt-tRNAs lead to mitochondrial dysfunction, fertility defects, embryonic and developmental arrest and, in a mutant lacking both types of modification, to robust lifespan extension. Our data show that there are important differences between the phenotypes of mutants lacking thiolation or carboxymethylaminomethylation including the expression pattern of both mitochondrial and nuclear genes. Our data are consistent with a model in which mitochondrial to nuclear retrograde signaling is different in different human mitochondrial translation diseases, and that these differences might help explain why clinical symptoms exhibited by people lacking the enzymes that modify positions 2 or 5 of U_34_ diverge.

## Results

### Characterization of the *C*. *elegans mttu-1*, *mtcu-1* and *mtcu-2* genes and their tRNA modification function

The *C*. *elegans* proteins encoded by the open reading frames (ORF) B0035.16 (CEOP4428 operon), F39B2.7 (CEOP1760 operon) and F52H3.2 (CEOP2696 operon) were respectively identified as the orthologues of TRMU, GTPBP3 and MTO1 using the protein Blast program (NCBI), and designated as MTTU-1 *(for nuclear encoded* ***m****itochondrial* ***t****RNA 2-****t****hio****u****ridylase)*, MTCU-1 and MTCU-2 *(for nuclear encoded* ***m****itochondrial* ***t****RNA* ***c****arboxymethyl-amino-methyl modification of* ***u****ridine residues*). Simplified diagrams of the three *C*. *elegans* genes are shown in S1A Fig. Sequence alignment revealed important conservation between the *C*. *elegans* proteins and their *E*. *coli*, yeast and human orthologues (S1 Table).

It is expected that MTTU-1, MTCU-1 and MTCU-2 localize to mitochondria like their yeast and human counterparts. To test experimentally whether the *C*. *elegans* genes include a mitochondrial targeting signal, the respective cDNAs were fused at their 3´ends to GFP and expressed in *Saccharomyces cerevisiae* under the *S*. *cerevisiae* GAL1 promoter. S1B Fig shows the subcellular co-localization of the GFP fusion proteins with the mitochondrial marker MitoTracker Red, confirming that the MTTU-1, MTCU-1 and MTCU-2 proteins localize to mitochondria in fungi, and that they can reach this subcellular compartment without resorting to a dedicated yeast mitochondrial targeting sequence.

In order to explore the molecular and cellular function of the *C*. *elegans mttu-1*, *mtcu-1* and *mtcu-2* genes, we made use of strains carrying *mttu-1*(*tm3160*), *mtcu-1*(*tm5041*) or *mtcu-2*(*ok2309*) deletion mutations. The mutations are predicted to disrupt the expression or function of the MTTU-1, MTCU-1 or MTCU-2 proteins since, in all cases, highly conserved sequences have been eliminated (S1A Fig). Worms homozygous for *mttu-1*(*tm3160*), *mtcu-1*(*tm5041*) or *mtcu-2*(*ok2309*) were viable and did not display any obvious defects in developmental patterning.

Our attempts to identify nucleosides cmnm^5^s^2^U, cmnm^5^U, and s^2^U by HPLC, UPLC or mass-spectrometry in mitochondrial RNA samples purified from the wild-type and mutant *C*. *elegans* strains were unsuccessful, likely because there were insufficient amounts of mt-tRNAs in our samples. Instead, therefore, we employed APM-Northern blotting to analyze the thiolation status of *C*. *elegans* ${\text{mt-tRNA}}_{\text{UAA}}^{\text{Leu}}$ and mt-tRNA^Gln^. This analysis relies on the increased retardation of thiolated molecules during gel electrophoresis in the presence of [p-(*N*-acryloylamino)-phenyl]mercuric chloride (APM). The thiol group has affinity for the compound in the gel and consequently migrates more slowly [30, 31]. The APM-Northern blotting technique is used regularly to estimate the thiolation status of mt-tRNAs from *TRMU*-deficient cells [8, 12, 17, 18, 28, 32, 33].

**Fig 2:**
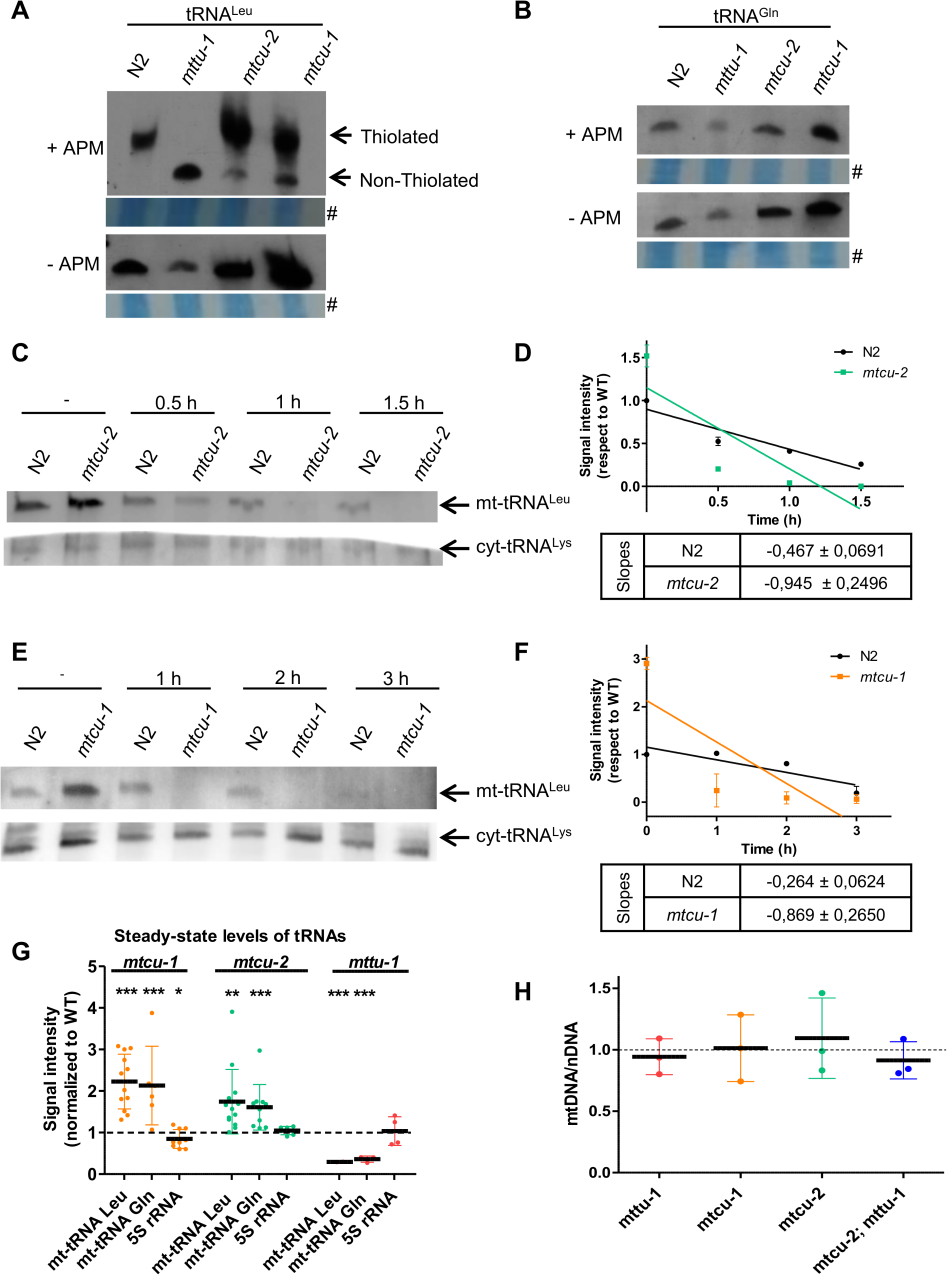
Effect of the *mttu-1*, *mtcu-1* and *mtcu-2* mutations on the modification status and steady-state levels of mt-tRNAs. (**A and B**) Analysis of the 2-thiolation status of ${\text{mt-tRNA}}_{\text{UAA}}^{\text{Leu}}$ (A) and mt-tRNA^Gln^ (B) by APM-Northern blotting. Total small RNAs obtained from mixed-stage populations of liquid-cultured worms were purified from wild-type (N2) and *mttu-1*, *mtcu-1* or *mtcu-2* strains, and analyzed in 10% polyacrylamide/8 M urea gels with (+) or without (-) 0.01 mg/ml APM. At least three replicates were performed. #Total small RNA stained with methylene blue. **(C-F)** Northern blot analysis of ${\text{mt-tRNA}}_{\text{UAA}}^{\text{Leu}}$ molecules after digestion with angiogenin *in vitro*. Three μg of total small RNA obtained from mixed-stage populations of liquid-cultured worms from N2 and *mtcu-2* (C) or *mtcu-1* (E) strains were digested with 12.5 μg/ml of angiogenin for the indicated times and ${\text{mt-tRNA}}_{\text{UAA}}^{\text{Leu}}$ and cyt-tRNA^Lys^ were detected with a specific probe. Quantification of at least two independent assays similar to those shown in C and E is given in D and F, respectively. **(G)** Quantification of the steady-state levels of ${\text{mt-tRNA}}_{\text{UAA}}^{\text{Leu}}$, mt-tRNA^Gln^ and 5S rRNA in *mttu-1*, *mtcu-1* and *mtcu-2* single mutants in comparison to the steady-state levels in wild-type strain (n≥3). **(H)** Quantification of mtDNA/nDNA ratio by qPCR. Four L4 worms grown at 20°C were used to quantify the mtDNA/nDNA ratio in each strain (n = 3). Error bars indicate ± SD (standard deviation). Statistical significance was evaluated with Student’s unpaired t-test. ** and *** denote p<0.01 and p<0.001, respectively.

${\text{mt-tRNA}}_{\text{UAA}}^{\text{Leu}}$ from the wild-type strain, but not from the *mttu-1* mutant, showed mobility retardation in the APM-containing gel (Fig 2A). This differential migration pattern indicates that the *mttu-1* gene is responsible for 2-thiouridylation in ${\text{mt-tRNA}}_{\text{UAA}}^{\text{Leu}}$. Most ${\text{mt-tRNA}}_{\text{UAA}}^{\text{Leu}}$ purified from strains carrying mutations in *mtcu-1* or *mtcu-2* migrated in the same way as ${\text{mt-tRNA}}_{\text{UAA}}^{\text{Leu}}$ purified from wild type (Fig 2A), indicating that these mutations do not strongly affect thiolation at position 2 of ${\text{mt-tRNA}}_{\text{UAA}}^{\text{Leu}}$. In contrast, no retardation was observed with mt-tRNA^Gln^ obtained from the wild-type and mutant strains, which means that this tRNA is natively non-thiolated; *i*.*e*. it is not a substrate for MTTU-1 (Fig 2B). According to these data, *C*. *elegans* ${\text{mt-tRNA}}_{\text{UAA}}^{\text{Leu}}$ and mt-tRNA^Gln^ exhibit identical U_34_ modification patterns at position 2 as their *A*. *suum* counterparts [28], and differ, therefore, from that found in *E*. *coli* tRNAs and mt-tRNA species of yeast and humans where the tRNA decoding for Gln, but not for Leu, is thiolated.

To analyze the involvement of MTCU-1 and MTCU-2 in tRNA modification, we used an assay based on the different sensitivity of hypomodified tRNAs to digestion by angiogenin [34, 35], a member of the RNase A superfamily. We have previously shown that *E*. *coli* tRNAs and human mt-tRNAs lacking modifications mediated by the MTCU-1 and MTCU-2 homologues are cleaved more efficiently by angiogenin than are the corresponding modified tRNAs [18]. Total small RNA isolated from N2 or the *mtcu-2* and *mtcu-1* single mutants was treated with angiogenin and the tRNA cleavage at different times was investigated by northern blot analysis using a specific probe for ${\text{mt-tRNA}}_{\text{UAA}}^{\text{Leu}}$ (Fig 2C and 2E). The kinetics of the nuclease digestion was obtained after quantification of the northern blot intensity (Fig 2D and 2F). We observed a higher sensitivity of ${\text{mt-tRNA}}_{\text{UAA}}^{\text{Leu}}$ towards digestion by angiogenin when small RNA was purified from the mutant strains (Fig 2C–2F), which suggests that *mtcu-1* and *mtcu-2* mutations alter the modification status of U_34_ at position 5.

It is noteworthy that ${\text{mt-tRNA}}_{\text{UAA}}^{\text{Leu}}$ and mt-tRNA^Gln^ appeared to be overrepresented in small RNA purified from the *mtcu-1* and *mtcu-2* mutants (Fig 2A, 2B, 2C and 2E), whereas they appeared to be underrepresented in small RNA purified from the *mttu-1* mutant (Fig 2A and 2B, -APM panels). Determination of the signal intensity produced by untreated ${\text{mt-tRNA}}_{\text{UAA}}^{\text{Leu}}$ and mt-tRNA^Gln^ in different northern blot assays of small RNA purified from the mutant and wild-type strains confirmed the accumulation of both mt-tRNAs in *mtcu-1* and *mtcu-2* mutants and their reduced expression in the *mttu-1* mutant (Fig 2G). These differences were not caused by the mtDNA copy number: determination of the mtDNA/nDNA ratio revealed no significant differences between any of the mutants and the wild-type strain (Fig 2H). Altogether these results suggest that loss of MTCU-1 or MTCU-2 promotes an adaptive response leading to increased synthesis or stability of, at least, ${\text{mt-tRNA}}_{\text{UAA}}^{\text{Leu}}$ and mt-tRNA^Gln^. In contrast, the adaptive response in the *mttu-1* strain leads to a decrease in the steady-state levels of these two mt-tRNAs. It should be noted that mt-tRNA^Gln^ is not a substrate for MTTU-1. Therefore, it appears that the adaptive mechanism triggered by the *mttu-1* mutation can affect mt-tRNAs that are not MTTU-1 substrates.

### Deletion of *mttu-1*, *mtcu-1* and *mtcu-2* results in mild OXPHOS dysfunction

**Fig 3:**
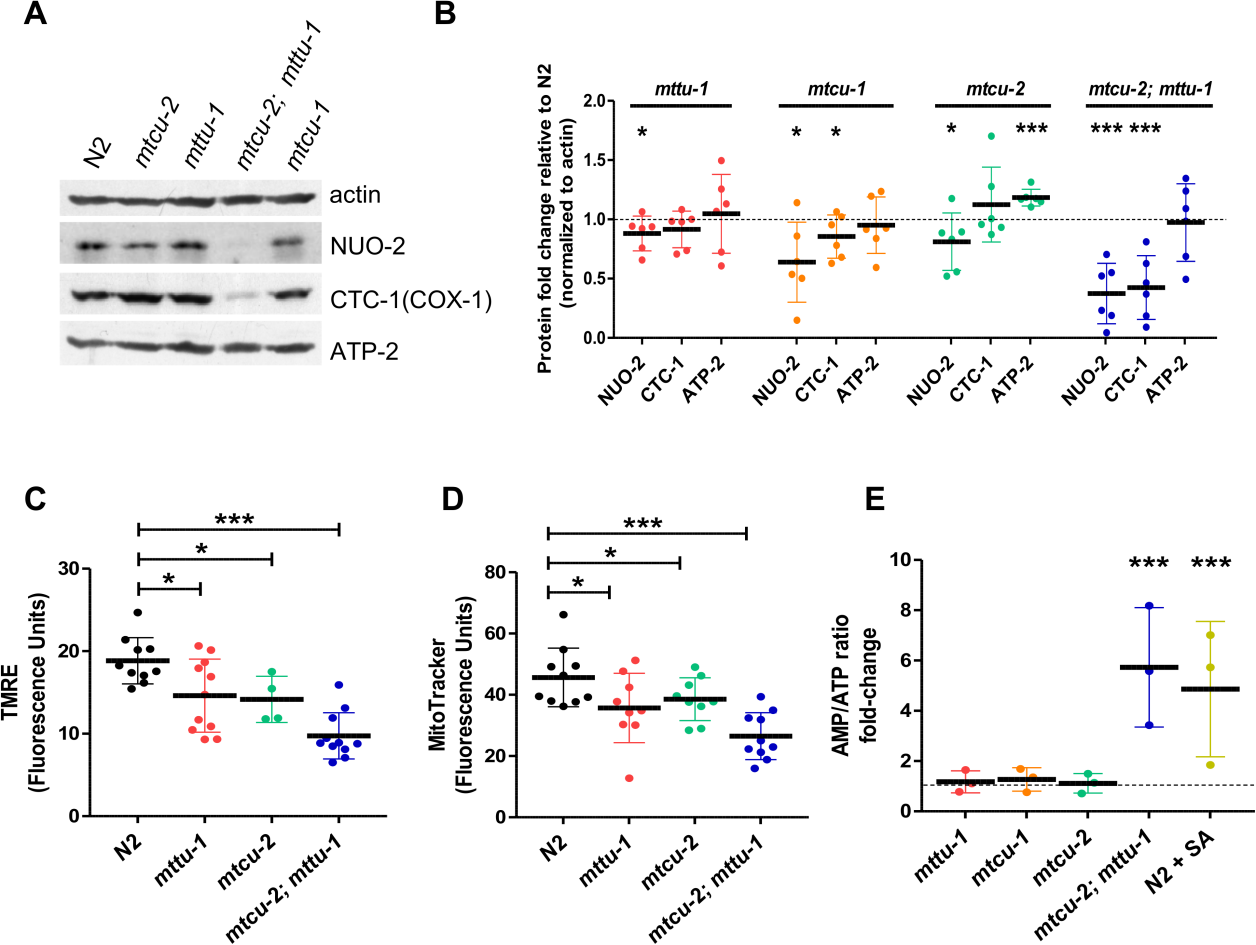
*mttu-1*, *mtcu-1* and *mtcu-2* mutants display mild mitochondrial defects. **(A)** Representative western blot of protein extracts from young adults worms (day 1 of adulthood) probed with antibodies to NUO-2 (complex I), CTC-1(COX-1) (complex IV), ATP-2 (complex V) and actin. **(B)** Densitometric analysis of blots obtained from at least three independent experiments. The steady state levels of the OXPHOS subunits were normalized with respect to actin and are represented as the equivalent amounts in wild type. Error bars indicate standard deviation (SD). * denotes p<0.05, ***, p = 0.0001. **(C, D)** Quantitation of (C) TMRE staining (n>4) (D) and MitoTracker Red staining (n>9) in L4 worms during 16 h. *p<0.05, ***p = 0.0002. **(E)** Graph showing the AMP/ATP ratio in the indicated strains at L4 stage. The wild-type strain (N2) treated for 2 h with 1 mM sodium azide (SA), which blocks ATP production, was included in the analysis as a positive control (n = 3). ***p<0.001. Statistical significance was evaluated with Student’s unpaired t-test. Data is represented as mean ± SD.

In humans, *TRMU*, *MTO1* and *GTPBP3* mutations cause dysfunction of the oxidative phosphorylation system by affecting the activity and steady-state levels of OXPHOS complexes, in particular those of complexes I and IV [8, 9, 11, 20]. We therefore investigated the effect of *mttu-1*, *mtcu-1* and *mtcu-2* mutations on different aspects of mitochondrial physiology. First, we explored the steady-state levels of the nuclear-encoded proteins NADH Ubiquinone Oxidoreductase NUO-2 (complex I) and ATP synthase subunit ATP-2 (complex V), and the mtDNA-encoded protein Cytochrome C Oxidase CTC-1(COX-1) (complex IV), which are homologous to human NDUFS3, ATP synthase subunit beta and COX1 respectively. The steady-state levels of NUO-2 were decreased slightly but significantly in all the single mutants compared to wild type (Fig 3A and 3B). Notably, there was a severe decrease in the levels of NUO-2 and COX-1 in the *mtcu-2; mttu-1* double mutant, suggesting a synergistic effect of the two mutations on biogenesis and/or stability of complex I and IV (Fig 3A and 3B). No decrease of the ATP-2 levels (complex V) was observed in either the double or single mutants (Fig 3A and 3B).

Next, we examined the mitochondrial membrane potential of the *mtcu-2* and *mttu-1* single mutants and the *mtcu-2; mttu-1* double mutant by staining with tetramethylrhodamine ethyl ester perchlorate (TMRE). Measurement and quantification of whole-worm TMRE fluorescence revealed a significant decrease of this parameter in the *mttu-1* and *mtcu-2* single mutants and a more drastic decrease, close to 50%, in the *mtcu-2; mttu-1* double mutant (Fig 3C). Analogous results were obtained with MitoTracker Red (Fig 3D).

Consistent with the severe effect on mitochondrial membrane potential, the AMP/ATP ratio was increased by more than 5-fold in the *mtcu-2*; *mttu-1* double mutant (Fig 3E). The AMP/ATP ratios were not significantly different from that in wild type in any of the single mutants. The increase in the ratio in the double mutant was not caused by lower mtDNA content, as determination of the mtDNA/nDNA ratio revealed no significant differences between the double mutant and the wild-type strain (Fig 2H).

**Fig 4:**
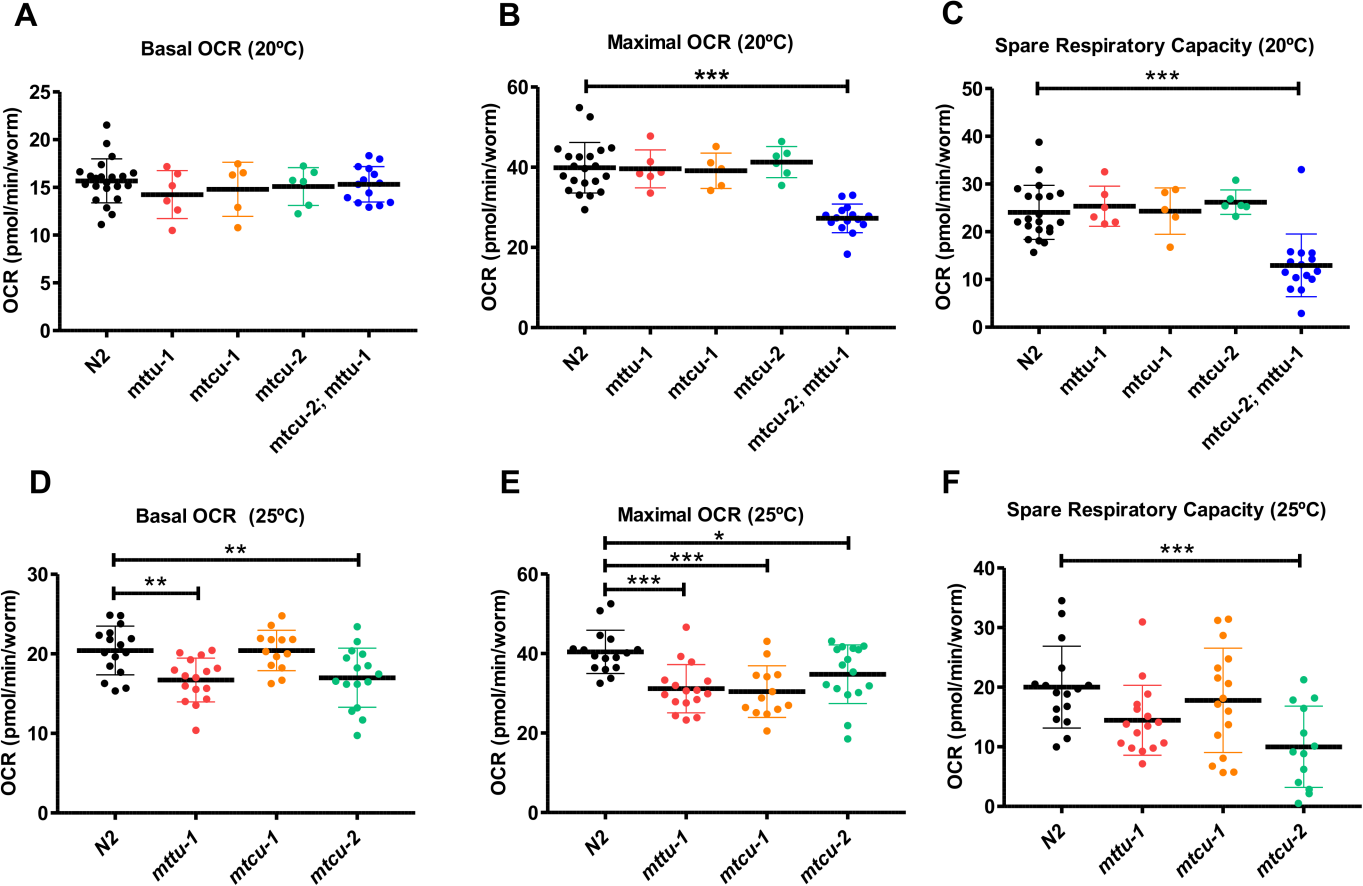
Mitochondrial respiratory capacity of the *mttu-1*, *mtcu-1*, *mtcu-2* and *mtcu-2; mttu-1* mutants. **(A-C)** Basal (A) and maximal (B) oxygen consumption rates and spare respiratory capacity (C) of worms grown at 20°C. Statistical significance for the double mutant was evaluated with unpaired T-test with Welch’s correction. *** denotes p<0.0001. **(D-F)** Basal (D) and maximal (E) oxygen consumption rates and spare respiratory capacity (F) of worms grown at 25°C. Statistical significance for the single mutants was evaluated with one-way ANOVA with a Dunnett’s post hoc test for multiple comparisons. *, ** and *** denote p<0.05, p<0.01 and p<0.001, respectively. Data is represented as mean ± SD.

We also investigated the impairment of the OXPHOS function in the mutants by monitoring the rates of oxygen consumption by live animals. No significant differences in basal and maximal oxygen consumption rate (OCR), or in spare respiratory capacity (SRC), (which indicates the organism’s ability to respond to increasing energy demands or other stress) were found between the wild-type strain and the single mutants at 20°C (Fig 4A–4C). In contrast, a significant decrease in the maximal OCR and SRC was observed between the wild-type strain and the double mutant under the same conditions (Fig 4A–4C). All three single mutants raised at 25°C had reduced maximal OCR compared to wild type (Fig 4E), and a decrease in the basal OCR was also detected in the *mttu-1* and *mtcu-2* mutants (Fig 4D). Under these conditions, a significant difference in the SRC respect to the wild type strain was only detected in the *mtcu-2* mutant (Fig 4F). Altogether, these results indicate that the single mutants have a mild OXPHOS dysfunction, which is mainly apparent under stress conditions, while the double mutant exhibits a clear OXPHOS dysfunction even under standard growth conditions.

**Fig 5:**
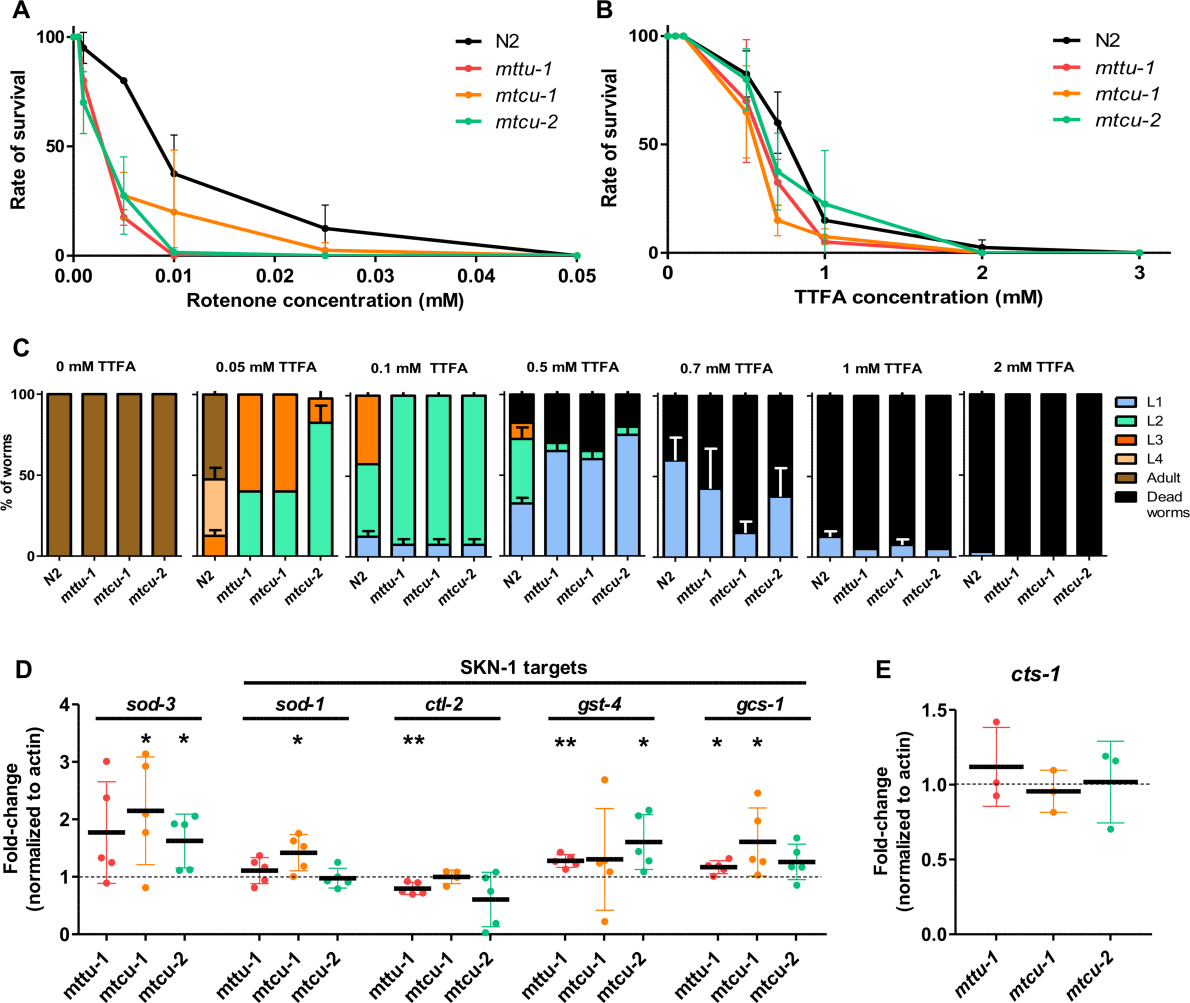
The single mutants exhibit higher sensitivity to inhibitors of complex I and II and mild antioxidant response. **(A, B)** Graphs showing survival of L1 worms after a 4-day exposure to different concentrations of the complex I inhibitor, rotenone (A) or the complex II inhibitor, TTFA (B) (n = 3). **(C)** Percentage of worms at different developmental stages and dead animals after a 4-day exposure to different concentrations of the complex II inhibitor TTFA. Treatment was initiated in synchronized L1 populations. **(D, E)** Quantitation of *sod-3*, *sod-1*, *ctl-2*, *gst-4*, *gcs-1* and *cts-1* mRNA levels by qRT-PCR in the indicated strains at L4 stage of development (n≥3). The mRNA levels were normalized to *act-1* and the wild-type strain. * denotes p<0.05, **, p<0.01 and ***, p<0.001. Statistical significance was evaluated with Student’s unpaired t-test and data is represented as mean ± SD.

Although the rates of oxygen consumption by the single mutants raised at 20°C were not significantly different from that by wild-type worms, the synergistic effect of *mtcu-2* and *mttu-1* mutations, the decrease in the steady-state levels of NUO-2 in the single mutants, and the decrease in the membrane potential observed in the *mttu-1* and *mtcu-2* mutants suggest that the OXPHOS system is partially impaired in the single mutants, likely at the level of complex I. It is possible that in the single mutants at 20°C a compensatory mechanism exists that allows respiration levels to be normal. For example, it is possible that in the single mutants under these conditions there is increased incorporation of FADH_2_-reducing equivalents into the OXPHOS system through complex II. To explore this hypothesis, we studied the effect of selective inhibitors of complex I and complex II on survival. A four-day treatment of first larval stage (L1) worms with rotenone, an inhibitor of complex I, caused growth arrest at L1 in both the wild-type strain and three single mutants. However, mutant larvae were more severely affected (Fig 5A). These results suggest that inhibition of complex I during L1 blocks larval development, and that complex I is partially impaired in the single mutants.

Although no differences were seen between the survival of mutant and wild-type strains upon treatment with TTFA, an inhibitor of complex II (Fig 5B), the rate of larval development was more strongly affected by the drug in the mutants than in wild type (Fig 5C). For example, at a TTFA concentration of 0.05 mM, about 50% of wild-type worms reached the adult stage, whereas none of the mutants did so. These data suggest that activity of complex II is particularly important for mutant larvae to be able to reach the L4/young adult stages. Synthesis of the complex II subunits (all of which are encoded by the nuclear DNA) should not be directly affected by the lack of MTTU-1, MTCU-1 or MTCU-2, since it occurs in the cytoplasm. Therefore, we suspect that the partial impairment of complex I in the single mutants is compensated by the activity of complex II, and that for this reason the TTFA-inhibition of this complex is especially detrimental for larval development of the mutants.

Since impairment of the OXPHOS function can lead to an increase in ROS production [1], we analyzed the mRNA expression of genes that are involved in the antioxidant response including *sod-1*, *ctl-2*, *gst-4*, and *gcs-1*, which are targets of SKN-1/Nrf2, a main regulator of a wide range of detoxification processes, and *sod-3*, which encodes an inducible mitochondrial superoxide dismutase [36–39]. With the exception of *ctl-2*, for all the genes analyzed there was a trend towards upregulation in the single mutants compared to the wild-type strain (Fig 5D). These results suggest that there is a moderate increase in ROS production in the single mutants, which slightly activates the antioxidant response.

We also analyzed the expression of the nuclear gene *cts-1* (which encodes mitochondrial citrate synthase) as a marker indicative of mitochondrial biogenesis. No significant differences were found between the single mutants and the wild-type strain (Fig 5E). These data, together with the mtDNA/nDNA ratio (Fig 2H), indicate that the changes that we observed in mt-tRNA levels (Fig 2G) and other mitochondrial parameters are not due to changes in mitochondrial abundance.

### Markers of the mitochondrial unfolding protein response are differentially expressed in *mttu-1* versus *mtcu-1* or *mtcu-2* mutants

**Fig 6:**
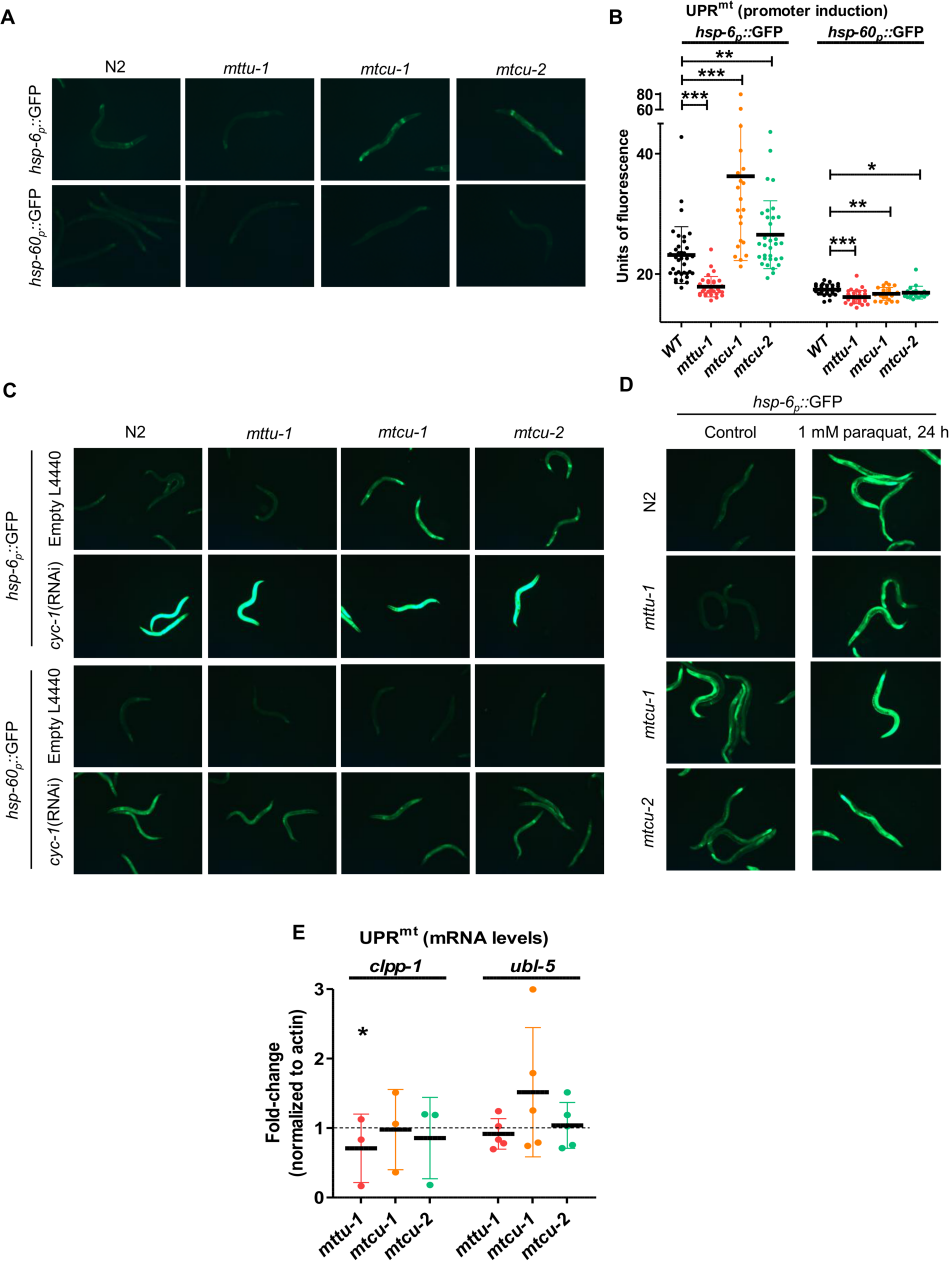
Expression of UPR^mt^ markers in *mttu-1*, *mtcu-*1 and *mtcu-2* mutant strains. **(A and B)** Expression of the *hsp-6*_*p*_::GFP or *hsp-60*_*p*_::GFP reporters in the indicated strains. Representative fluorescence micrographs of wild-type and single mutants harbouring *hsp-6*_*p*_::GFP or *hsp-60*_*p*_::GFP transgenes at L4 stage of development are shown in (A). Quantification is shown in (B) (n>20). * denotes p<0.05, **, p<0.01 and ***, p<0.001. **(C)** Fluorescence micrographs of wild-type and single mutants harbouring *hsp-6*_*p*_::GFP or *hsp-60*_*p*_::GFP transgenes at L4 stage of development after *cyc-1*(RNAi) from the L1 stage. **(D)** Fluorescence micrographs of one-day old, adult wild type and single mutants harbouring *hsp-6*_*p*_::GFP transgene exposed to 1 mM paraquat for 24 h. Note that *cyc-1*(RNAi) (C) and paraquat treatment (D) cause marked increases in GFP expression. **(E)** Quantitation of *clpp-1* and *ubl-5* mRNA levels by qRT-PCR in the indicated strains at L4 stage (n≥3). The mRNA levels were normalized to those of *act-1* in the wild-type strain. Statistical significance was evaluated with Student’s unpaired t-test. Error bars indicate standard deviation (SD). *, p<0.05.

The reduction in the stationary levels of NUO-2, a nuclear encoded OXPHOS protein, in *mtcu-1*, *mtcu-2*, and *mttu-1* (Fig 3A and 3B) suggests that the synthesis and/or assembly of complex I subunits may be impaired in these mutants. Loss of mitochondrial protein homeostasis due to incorrectly synthesized mtDNA-encoded proteins and degradation of unfolded or unassembled mitochondrial proteins results in the activation of the mitochondrial unfolded response or UPR^mt^ [40–42]. In *C*. *elegans*, the UPR^mt^ is associated with strongly increased expression of *hsp-6* and *hsp-60* (which encode mitochondrial chaperones homologous to human mt-HSP70 and mt-HSP60, respectively). Worm strains expressing a GFP reporter fused to the *hsp-6* and *hsp-60* promoters (*hsp-6*_*p*_::GFP and *hsp-60*_*p*_::GFP, respectively) are frequently used to evaluate the activation of the UPR^mt^. Therefore, we analyzed the effect of the *mttu-1*, *mtcu-1* and *mtcu-2* mutations on the expression of *hsp-6*_*p*_::GFP and *hsp-60*_*p*_::GFP reporters. Strikingly, expression of *hsp-6*_*p*_::GFP was down-regulated in the *mttu-1* mutant, but up-regulated in the *mtcu-1* and *mtcu-2* mutants, whereas expression of *hsp-60*_*p*_::GFP was slightly, but significantly, down-regulated in all three mutants (Fig 6A and 6B).

The differential expression of the *hsp-6*_*p*_::GFP reporter in the *mtcu-1* and *mtcu-2* strains versus the *mttu-1* strain (Fig 6B, left) is particularly interesting since it suggests that loss of the *mtcu-1* or *mtcu-2* function generates a mitochondrial stress that is different from that triggered by the loss of the *mttu-1* function, leading to different nuclear responses. It is worth noting that the constitutive induction of the *hsp-6*_*p*_::GFP reporter in the *mtcu-1* and *mtcu-2* single mutants was not affected by antioxidant (N-Acetyl-L-cystein, NAC) treatment (S2 Fig), suggesting that oxidative stress is not the main cause of the induction of the *hsp-6* promoter in these strains.

To determine whether the *hsp-6* and *hsp-60* promoters can be induced in the single mutants by treatments classically used to induce the UPR^mt^, worms carrying the *hsp-6*_*p*_::GFP or *hsp-60*_*p*_::GFP reporters were silenced from the first stage (L1) of development with the *cyc-1*(RNAi) clone. Induced expression of both reporters was observed in all single mutants (Fig 6C). Moreover, paraquat treatment (1 mM for 24 h) also induced the expression of the *hsp-6*_*p*_::GFP reporter in the single mutants (Fig 6D). Altogether these data indicate that the *mttu-1*, *mtcu-1* and *mtcu-2* mutations do not inhibit the induction of the *hsp-6* and *hsp-60* promoters that occurs through the classically defined UPR^mt^ pathway.

In *C*. *elegans*, UPR^mt^ induction requires CLPP-1, a mitochondrial protease, and UBL-5, an ubiquitin-like protease. CLPP-1 acts as a sensor of the UPR^mt^ by recognizing and degrading accumulated unfolded proteins; UBL-5 is thought to cooperate with the main UPR^mt^ transcriptional regulator, ATFS-1, to activate chaperone expression [40–44]. We examined *clpp-1* and *ubl-5* levels by qRT-PCR. *clpp-1* was found to be down-regulated (~25%) in the *mttu-1* single mutant but expressed at nearly normal levels in *mtcu-1* and *mtcu-2* (Fig 6E). No significant changes were seen in the expression of *ubl-5* (Fig 6E).

In brief, our findings support the proposal that the MTTU-1 defect leads to a nuclear response different from that caused by the MTCU-1 or MTCU-2 defect, as the *hsp-6* promoter expression is different in each type of mutant (Fig 6B).

### Inactivation of *mttu-1*, *mtcu-1* or *mtcu-2* induces changes in the expression pattern of metabolism genes

Dysregulation of genes encoding enzymes involved in intermediary metabolism has been reported from cell and murine models of the GTPBP3 and TRMU defects respectively [18, 21]. Therefore, we examined the mRNA expression of genes relevant for metabolic processes related to mitochondrial function.

**Fig 7:**
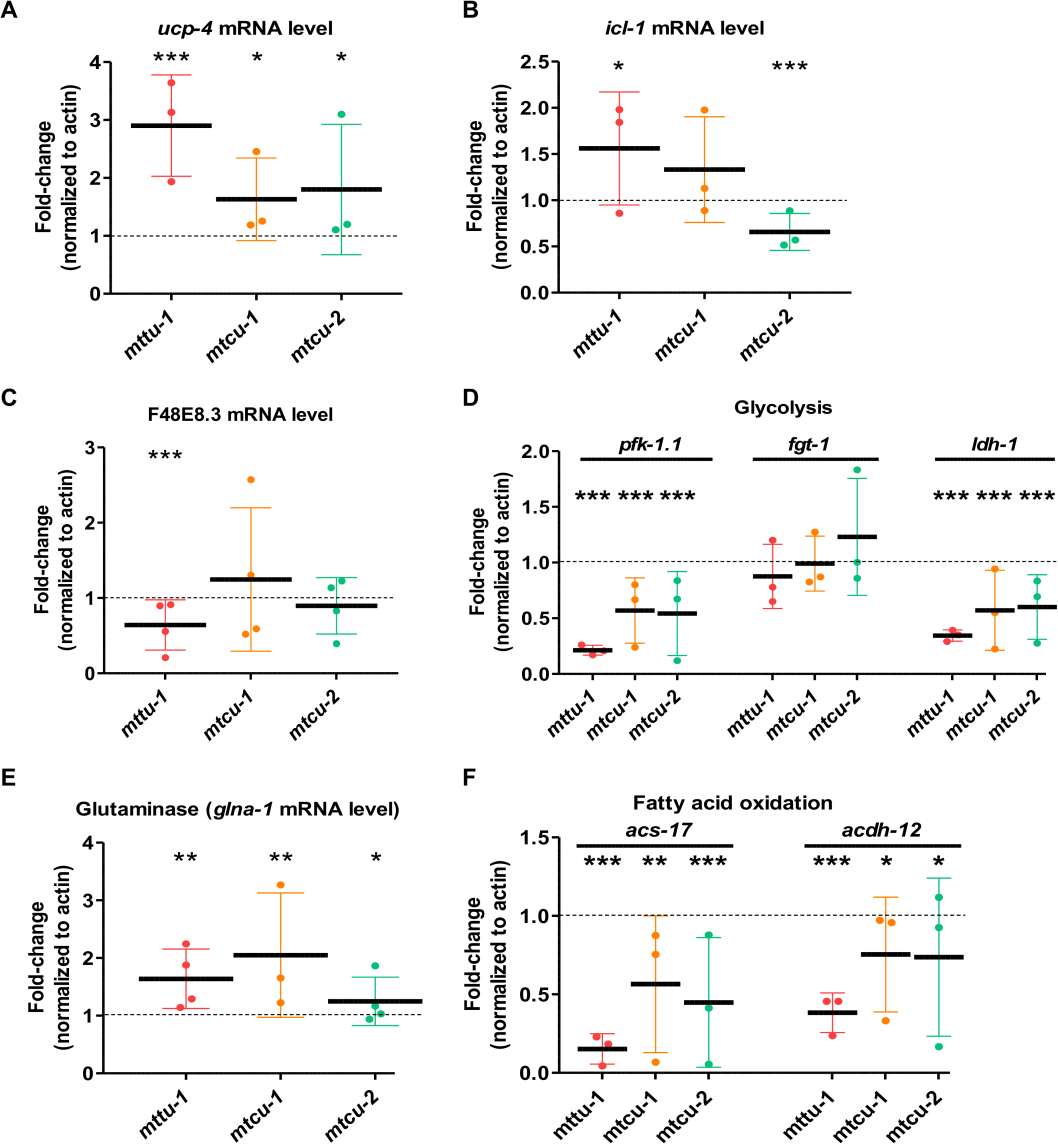
mRNA expression of metabolic genes in *mttu-1*, *mtcu-1* and *mtcu-2* single mutants. (**A-F**) qRT-PCR analysis of mRNA expression of genes related to 1) succinate import to mitochondria (*ucp-4*, panel A), 2) glyoxylate cycle (*icl-1*, panel B), 3) malate dismutation **(**F48E8.3, panel C), 4) glycolysis (*pfk-1*.*1*, *fgt-1* and *ldh-1*, panel D), 5) glutaminolysis (*glna-1*, panel E), and 6) fatty acid oxidation (*acs-17* and *acdh-12*, panel F) in synchronized L4 populations. The mRNA levels of the selected genes were normalized to those of *act-1* in the wild-type strain (n≥3). * denotes p<0.05, **p<0.01 and ***p<0.001. Statistical significance was evaluated with Student’s unpaired t-test.

It has been shown that UCP-4, the only member of the mitochondrial uncoupling protein family present in *C*. *elegans*, regulates the function of complex II by controlling import of succinate, the direct substrate of complex II, into mitochondria [45]. Interestingly, we observed approximately 3- and 0.5-fold increases of the *ucp-4* mRNA levels in the *mttu-1* and *mtcu-1* or *mtcu-2* single mutants, respectively (Fig 7A).

Two putative sources of succinate outside mitochondria are the glyoxylate cycle and malate dismutation. Nematodes, unlike mammals, have been shown to possess an active glyoxylate shunt which converts isocitrate to succinate and malate using glyoxylate as an intermediate [46, 47]. This cycle allows cells to convert two acetyl-CoA units generated by various catabolic processes into C4-units (succinate) which can be used to replenish the TCA cycle or to function as precursors for amino acid or carbohydrate biosynthesis [47]. In *C*. *elegans*, the key enzymatic activities, isocitrate lyase and malate synthase, are in two separate structural domains of a single protein encoded by the *icl-1* gene [48]. The subcellular location of ICL-1 is controversial [49, 50], and it cannot be ruled out that the protein is localized in both cytoplasm and mitochondria. We analyzed the mRNA expression of the *icl-1* gene in the single mutants and found it was increased by about 50% in *mttu-1*, but unchanged or slightly decreased in the *mtcu-1* and *mtcu-2* mutants (Fig 7B). These data suggest that there may be increased activity of the glyoxylate shunt in the *mttu-1* mutant but not in the *mtcu-1* and *mtcu-2* mutants.

The soluble fumarase reductase activity encoded by the F48E8.3 gene is part of the malate dismutase mechanism and produces succinate from fumarate in the cytosol of *C*. *elegans* cells [51]. We measured the F48E8.3 mRNA levels in all three single mutants and found they were reduced by about 35% in the *mttu-1* mutant but were not significantly altered in the *mtcu-1* and *mtcu-2* mutants in comparison to the wild-type strain (Fig 7C).

In order to explore the putative contribution of genes involved in glycolysis, fatty acid oxidation and glutaminolysis to changes in the TCA cycle in the single mutants, we analyzed the mRNA expression of genes encoding key enzymes controlling these processes. To study the implication of glycolysis in the OXPHOS functioning, we analyzed the mRNA levels of *pfk-1*.*1*, *fgt-1* and *ldh-1*. The *pfk-1*.*1* gene encodes phosphofructokinase 1, a rate-limiting enzyme in glycolysis. *fgt-1* encodes a glucose transmembrane transporter and *ldh-1* encodes lactate dehydrogenase, which is responsible for generation of lactate from pyruvate. Although no change was observed in the mRNA levels of *fgt-1*, a significant decrease in the expression of both *pfk-1*.*1* and *ldh-1* was seen in all three single mutants, suggesting that glycolysis is reduced in all three single mutants (Fig 7D).

GLNA-1 is the *C*. *elegans* ortholog of human GLS1 and GLS2 and thus predicted to have glutaminase activity and to transform glutamine to glutamate, which in turn can be transformed in α-ketoglutarate, an intermediate of the TCA cycle. We found that the *glna-1* mRNA levels were significantly increased in all the single mutants (Fig 7E).

Finally, we analyzed the expression of two genes involved in the fatty acid oxidation pathway, *acs-17* and *acdh-12*, which encode long-chain acyl-CoA synthetase, and very long chain acyl-coA dehydrogenase, respectively. Both genes were found to be down-regulated in all three single mutants (Fig 7F), suggesting that fatty acid oxidation is reduced in these mutants.

Taken together, our data indicate that the expression of metabolism genes is appreciably altered in the single mutants compared to the wild-type strain, and that the expression profile of the *mttu-1* mutant exhibits significant differences from that of the *mtcu-1* and *mtcu-2* mutants.

### *mttu-1*, *mtcu-1* and *mtcu-2* mutations cause defects in fertility and in the reproductive cycle

**Fig 8:**
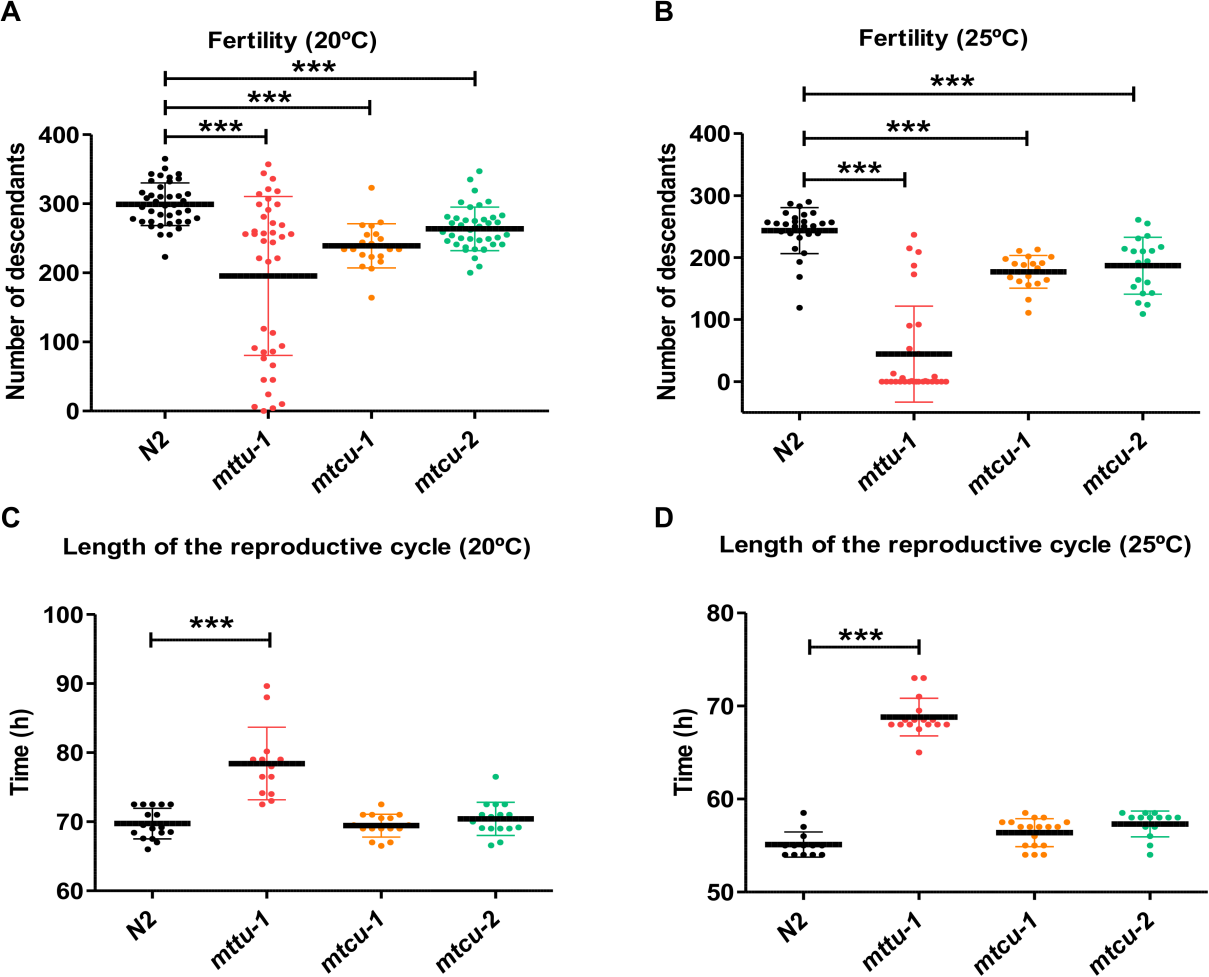
Effect of the *mttu-1*, *mtcu-1* and *mtcu-2* mutations on fecundity and reproductive cycle length. **(A, B)** Fertility of the wild-type (N2), *mttu-1*, *mtcu-1* and *mtcu-2* strains was measured by the number of progeny laid by adult hermaphrodite worms at 20°C (A) and 25°C (B) (n≥2). **(C, D)** Length of the reproductive cycle in the wild-type, *mttu-1*, *mtcu-1* and *mtcu-2* strains at 20°C (C) and 25°C (D) (n≥3). *** denotes p<0.001. Statistical significance was evaluated with Student’s unpaired t-test.

The *mttu-1*, *mtcu-1* and *mtcu-2* single mutant strains can all be propagated as homozygotes. We found, however, that all three had reduced fertility and that the *mttu-1* mutants also grew more slowly (Fig 8). Both the fertility and slow growth defects of the *mttu-1* mutants were temperature sensitive (Fig 8B and 8D). Loss of *mttu-1* activity had a greater effect on fertility and reproductive cycle than the loss of *mtcu-1* or *mtcu-2*. With the caveat that the *mtcu-1* mutation used also affects the neighboring gene (F39B2.5), the data also show that the phenotypes of the *mtcu-1* and *mtcu-2* mutants are rather similar.

**Fig 9:**
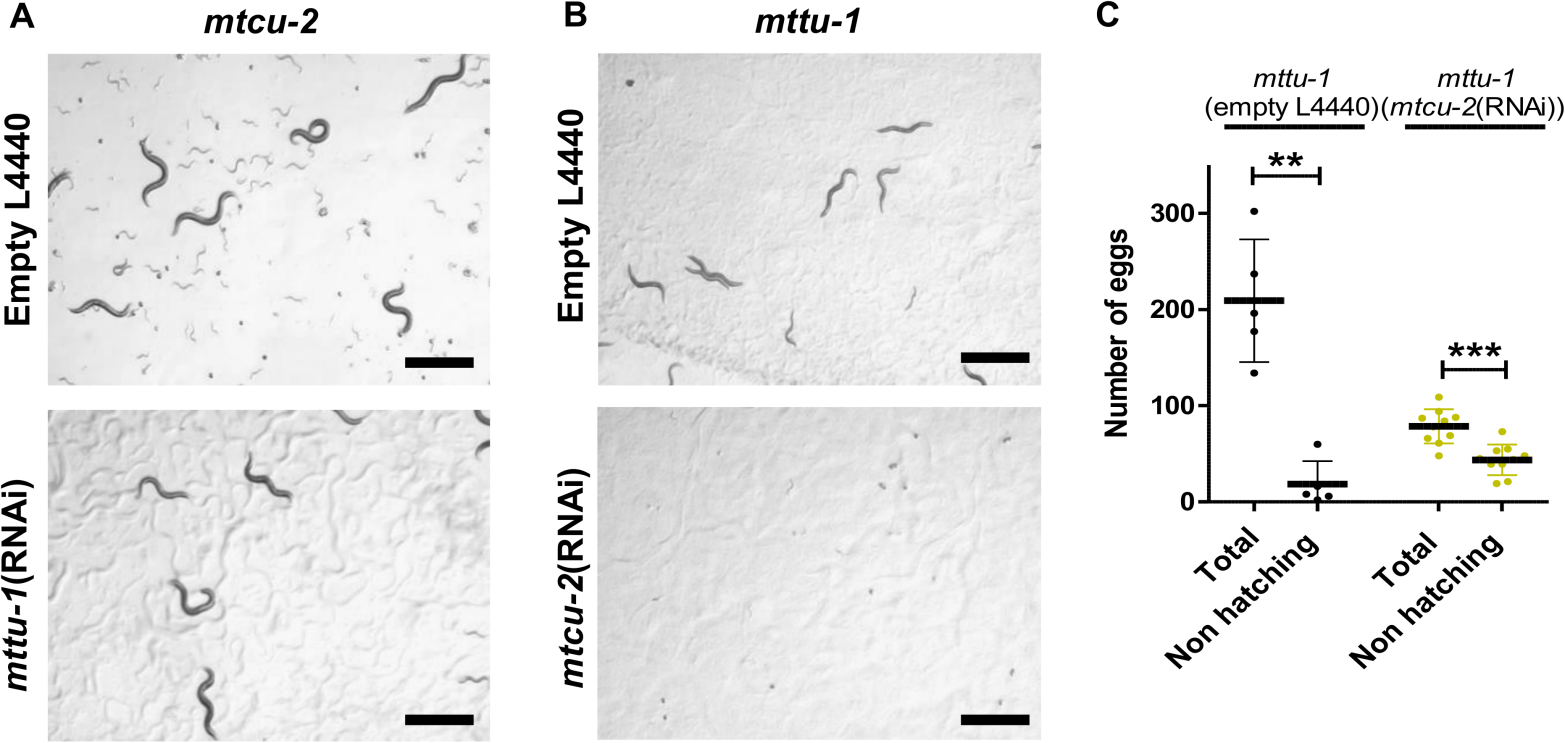
Simultaneous lack of mitochondrial MTTU-1 and MTCU-2 proteins is associated with embryonic lethality, developmental defects and sterility. **(A)** Silencing of *mttu-1* in the *mtcu-2* mutant (from L4 stage onwards) at 25°C produces a slower rate of development and sterility of their progeny. **(B)** and **(C)** Silencing of *mtcu-2* in the *mttu-1* mutant (from L4 stage onwards) at 25°C causes arrest of development at the L1-L2 stages (B) and embryonic lethality (C). The total number of eggs and the number that failed to hatch were quantified (n≥5). **p<0.01, ***p<0.001. Statistical significance was evaluated with Student’s unpaired t-test. Error bars indicate standard deviation (SD).

In both *E*. *coli* and yeast, the simultaneous inactivation of the *mttu-1* and *mtcu-1* or *mtcu-2* orthologues produces synthetic lethality under growth conditions in which each gene, separately, is dispensable [17, 32, 33]. We found that the *C*. *elegans* genes also showed marked synthetic defects. In particular, most *mtcu-2*; *mttu-1* double mutant embryos segregating from mothers that were heterozygous for *mttu-1* failed to hatch. Those that did hatch developed into adults that were invariably completely sterile. *mtcu-2*; *mttu-1*(RNAi) worms also grew more slowly than wild type and were frequently sterile (Fig 9A, S3 Fig). More than 50% of *mtcu-2*(RNAi); *mttu-1* embryos failed to hatch and larvae arising from those eggs that did invariably arrested development at early larval stages (Fig 9B and 9C, S4 Fig). Thus, the simultaneous inactivation of the *mttu-1* and *mtcu-2* genes drastically affects multiple stages of *C*. *elegans* development. The results also suggest that the ability of some *mtcu-2; mttu-1* double mutants to develop into adults is likely the result of partial maternal rescue. It is possible that the MTTU-1 or MTCU proteins themselves are segregated into embryos from their mothers. Alternatively, the tRNAs they modify may themselves be relatively stable.

**Fig 10:**
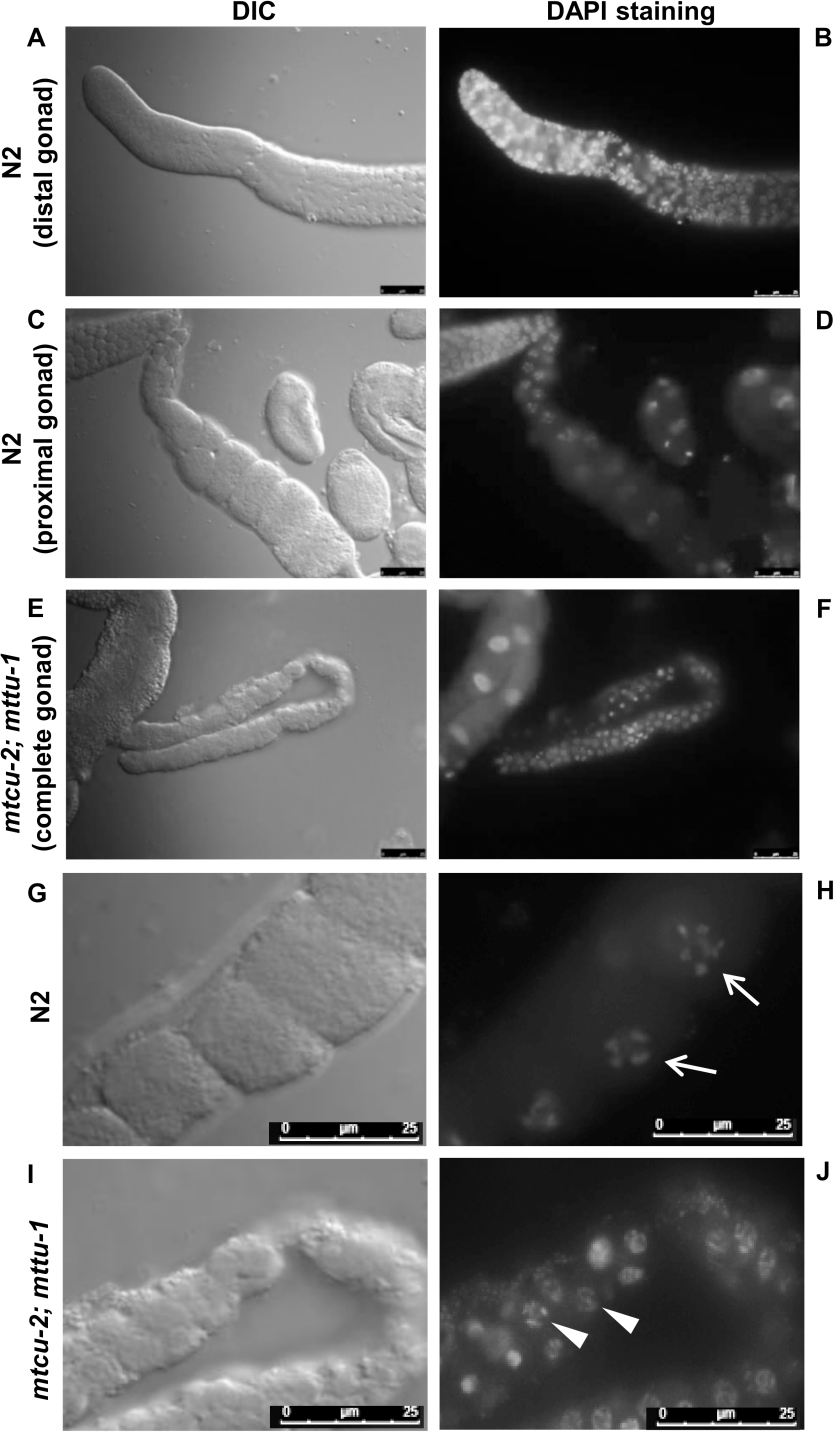
The simultaneous lack of mitochondrial MTTU-1 and MTCU-2 proteins causes gonadal defects. (**A, C, E, G and I**) Nomarski differential contrast (DIC) and (**B, D, F, H and J**) fluorescence micrographs of DAPI-stained dissected gonads from N2 wild-type and *mtcu-2; mttu-1* double mutant hermaphrodites. Distal (A and B) and proximal (C and D) gonad from a wild-type hermaphrodite, as well as a complete gonad from an *mtcu-2; mttu-1* double mutant (E and F) are shown. (**G-J**) Detail of the proximal region of gonads from N2 and *mtcu-2; mttu-1* strains. In N2 worms, germ cells in this region are in diplotene and bivalents (white arrows) are visible in cells progressing to form oocytes, which are arrested at diakinesis. In the *mtcu-2; mttu-1* double mutant in contrast, no bivalents or mature oocytes were visible (the white arrowheads indicate germ cell nuclei arrested in diplotene). Scale bar: 25 μm. Pictures shown in A to F were taken at the same magnification as were those in G to J.

To investigate the sterility defect of the *mtcu-2; mttu-1* double mutant hermaphrodites, we stained gonads with DAPI. These experiments indicated that there was a clear defect from the L4 stage onwards in the germline itself: the gonads were considerably smaller in size (Fig 10, compare A and C with E) probably because they contained fewer germ cells than wild-type gonads (Fig 10, compare B and D with F). Moreover, both sperm and mature oocytes were absent (Fig 10, compare H and J). Besides reduced numbers of germline cells, other germline defects observed included a marked increase in the number of cells arrested with 6 bivalents (Fig 10J).

### *mtcu-2*; *mttu-1* double mutants have increased longevity

**Fig 11:**
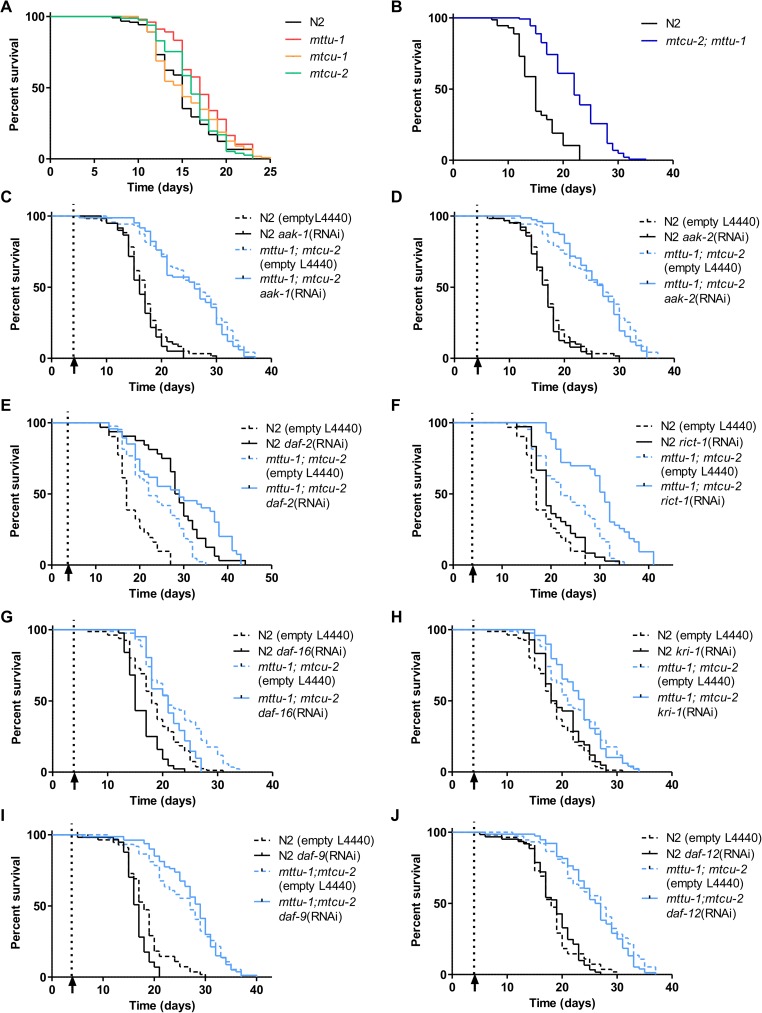
Simultaneous inactivation of MTTU-1 and MTCU-2 leads to lifespan extension in *C*. *elegans*. **(A)** Survival of the wild-type strain and the *mttu-1*, *mtcu-1* and *mtcu-2* single mutants at 20°C (n = 4). **(B)** Survival of the wild-type strain and the *mtcu-2; mttu-1* double mutant at 20°C (n = 3). **(C-J)** *aak-1* (n = 2) (C), *aak-2* (n = 2) (D), *daf-2* (n = 1) (E), *rict-1* (n = 1) (F), *daf-16* (n = 2) (G), *kri-1* (n = 2) (H), *daf-9* (n = 2) (I), and *daf-12* (n = 2) (J) silencing effect on the survival of the N2 and *mtcu-2; mttu-1* strains at 20°C. The empty vector L4440 was used as a negative control. Animals used for controls were of the same age as the experimental animals. To avoid disturbing embryonic development, silencing was started at the L4 stage (pointed with and arrow and a dashed line). Statistical significance was evaluated with Log-rank (Mantel Cox test) and Gehan-Breslow-Wilcoxon test and statistics are shown in Table 1.

In *C*. *elegans*, a correlation among OXPHOS dysfunction, sterility, and lifespan extension has been observed in certain mutants [52, 53]. Therefore, we compared the lifespan of the *mttu-1*, *mtcu-1* and *mtcu-2* single mutants and the *mtcu-2; mttu-1* double mutant with that of the wild-type strain. As shown in Fig 11A and 11B and Table 1, lifespan of the single mutants was slightly extended at 20°C, but the increase was only statistically significant for the *mttu-1* strain. Both the median and maximum lifespan of the *mtcu-2*; *mttu-1* double mutant were extended by about 50% indicating that the simultaneous inactivation of *mttu-1* and *mtcu-2* has an important impact on longevity in *C*. *elegans*. These findings prompted us to perform a preliminary study of some longevity pathways in the *mtcu-2*; *mttu-1* double mutant.

**Table 1:** Effect of the genetic background and RNAi silencing on *C*. *elegans* lifespan at 20°C.

Strain	RNAi	Median life^#^ (days)	Max. life^##^ (days)	Death/ Total	Log-rank (Test Mantel Cox)^$^ p-value	Test Gehan-Breslow-Wilcoxon ^$^ p-value
N2	-	15	23	116/127	-	-
*mttu-1*	*-*	17	23	98/115	***<0,0001	***<0,0001
*mtcu-1*	*-*	15	25	112/124	ns	ns
*mtcu-2*	*-*	16	23	78/91	ns	*0,0169
N2	*-*	15	23	67/73	***<0,0001	***<0,0001
*mtcu-2; mttu-1*	*-*	22	35	144/146		
N2	Negative control	17	30	57/60	ns	ns
	*aak-1*	16	24	59/60		
*mtcu-2; mttu-1*	Negative control	27	37	71/71	ns	ns
	*aak-1*	26	37	82/84		
N2	Negative control	17	30	57/60	ns	ns
	*aak-2*	17	25	61/64		
*mtcu-2; mttu-1*	Negative control	27	37	71/71	ns	ns
	*aak-2*	27	35	75/81		
N2	Negative control	17	27	31/33	***<0,0001	***<0,0001
	*daf-2*	22	41	32/33		
*mtcu-2; mttu-1*	Negative control	28	35	43/43	***0,0002	*0,0179
	*daf-2*	29	43	41/47		
N2	Negative control	17	27	31/33	*0,0364	*0,0243
	*rict-1*	19	34	36/37		
*mtcu-2; mttu-1*	Negative control	22	35	43/43	***<0,0001	***<0,0001
	*rict-1*	31	41	43/43		
N2	Negative control	18	31	81/81	***0,0001	***0,0007
	*daf-16*	15	24	41/44		
*mtcu-2; mttu-1*	Negative control	21	34	84/85	*0,0178	ns
	*daf-16*	21	27	41/41		
N2	Negative control	18	31	81/81	ns	ns
	*kri-1*	18	28	42/46		
*mtcu-2; mttu-1*	Negative control	21	34	84/85	ns	ns
	*kri-1*	24	34	49/50		
N2	Negative control	18	30	55/59	**0,0013	*0,013
	*daf-9*	17	21	57/58		
*mtcu-2; mttu-1*	Negative control	27	37	74/74	ns	ns
	*daf-9*	29	40	80/81		
N2	Negative control	18	30	55/59	ns	ns
	*daf-12*	19	27	61/62		
*mtcu-2; mttu-1*	Negative control	27	37	74/74	ns	ns
	*daf-12*	26	37	76/77		

Median (#) and maximum (##) life span as well as statistics ($) of each strain and condition are summarized in the table.

Non-significant (ns).

* denotes p<0.05

** p<0.01

*** p<0.001

One of the mechanisms underlying lifespan extension caused by OXPHOS dysfunction involves the energy sensing AMP-activated protein kinase, AMPK [23, 53–55]. Knocking down the expression of the two homologues of AMPKα present in *C*. *elegans* (encoded by *aak-1* and *aak-2* [56]) had no effect on the lifespan of wild-type and *mtcu-2; mttu-1* worms. While preliminary data suggested that *aak-1* is not required for the control of lifespan [56], which would be in agreement with our data (Fig 11C, Table 1), *aak-2*(*ok524*) mutants were reported to have about 15–25% shorter lifespan than wild-type animals [56, 57]. Given that downregulation of *aak-2* by RNAi had no effect on lifespan of the wild-type worms (Fig 11D, Table 1), it is unclear from our data whether AMPK participates in the longevity of the *mtcu-2; mttu-1* double mutant.

Besides being affected by changes in mitochondrial function, longevity in *C*. *elegans* is also regulated by a number of different conserved signaling pathways including an insulin/insulin-like (IIS) pathway, the Target of Rapamycin (TOR) pathway, the germline pathway and the steroid hormone pathway.

The IIS pathway prevents translocation of the FOXO transcription factor DAF-16 to the nucleus, whereas the TORC2 complex, which includes protein RICT-1, impairs the activity of the transcription factor SKN-1 [58]. Knockdown of *daf-2* (which encodes the insulin receptor acting at the beginning of the IIS pathway) or *rict-1* extended the lifespan of the *mtcu-2*; *mttu-1* double mutant (Fig 11E and 11F, Table 1) indicating that lack of *mtcu-2* and *mttu-1* does not extend lifespan solely by reducing the activity of the IIS or TORC2 pathways. Notably, knockdown of *daf-16* itself had only a minor decreasing effect on lifespan of the *mtcu-2*; *mttu-1* double mutant (Fig 11G, and Table 1).

The KRI-1/SKN-1/UPR^mt^-dependent pathway, which links reproductive signaling to mitochondria, plays a key role in the process by which ROS promote life-extension in respiratory-chain mutants [59]. Knockdown of *kri-1* did not affect the extended lifespan of the *mtcu-2*; *mttu-1* strain (Fig 11H, Table 1) suggesting that *kri-*1 does not play an essential role in the longevity of this mutant.

Two major components of the steroid signaling pathway are DAF-12, a nuclear hormone receptor, and DAF-9, a cytochrome P450 involved in the synthesis of DAF-12 ligands (dafachronic acids) [60–63]. Both the ligand-bound and unbound forms of DAF-12 affect longevity but can do so in different ways depending upon the context [61, 63–66]. We found that the increased longevity of the *mtcu-2*; *mttu-1* double mutant was epistatic to the decreased longevity caused by *daf-9*(RNAi), a result that would be consistent with a model in which MTCU-2 and MTTU-1 affect longevity through DAF-12 (Fig 11I, and Table 1). However, *daf-12*(RNAi) did not have any significant effect on the increased longevity of the *mtcu-2*; *mttu-1* double mutant indicating that MTCU-2 and MTTU-1 cannot act solely through DAF-12 (Fig 11J, and Table 1).

Together, these results suggest that the increased longevity of the *mtcu-2*; *mttu-1* double mutant is the result of effects on several different pathways. The identification of the mechanisms involved in longevity of the *mtcu-2; mttu-1* double mutant stands to be an exciting topic for future investigation.

## Discussion

We present here *C*. *elegans* models for human diseases associated with hypomodification at position 2 and 5 of U_34_ in a mt-tRNA set. Consistent with the observation that lack of modification at the 2 and 5 positions causes very different clinical symptoms (liver failure (TRMU) or hypertrophic cardiomyopathy (GTPBP3 and MTO1) respectively), we have demonstrated that the phenotype of the mutant lacking the modification at position 2 (*mttu-1*) of U_34_ is appreciably different from that of mutants lacking the modification at position 5 (*mtcu-1* or *mtcu-2*). The differences include those in the pattern of expression of nuclear and mitochondrial genes. We propose that the different hypomodification defects trigger specific changes in retrograde signaling that account for differences in phenotype at the organismal level.

We have shown that the *C*. *elegans mttu-1* gene controls thiolation of ${\text{mt-tRNA}}_{\text{UAA}}^{\text{Leu}}$, and that the *mtcu-1* and *mtcu-2* defects increase the sensitivity of ${\text{mt-tRNA}}_{\text{UAA}}^{\text{Leu}}$ to angiogenin digestion, a feature also observed in bacterial tRNAs lacking the modification catalyzed by the MTCU-1 and MTCU-2 homologues [18]. Therefore, we conclude that, as expected, *mttu-1*, *mtcu-1* and *mtcu-2* have similar roles to those of their orthologues in *E*. *coli*, yeast and humans. Our data also indicate that the modification at position 2 by MTTU-1 is independent of MTCU-1 and MTCU-2 since the inactivation of these proteins does not affect the thiolation status of ${\text{mt-tRNA}}_{\text{UAA}}^{\text{Leu}}$. Independence of the modification pathways controlled by the MTTU-1 and MTCU-1/MTCU-2 homologues has been previously demonstrated in *E*. *coli* and *S*. *cerevisiae* [32, 67, 68], and more recently in GTPBP3-silenced cells [18] and in a liver-specific Mtu1-deficient mouse [21]. In addition, our results suggest that the *C*. *elegans* mt-tRNAs follow the modification pattern of the *A*. *suum* mt-tRNAs [28] and not that of yeast and human mt-tRNAs, since ${\text{mt-tRNA}}_{\text{UAA}}^{\text{Leu}}$ is thiolated but mt-tRNA^Gln^ is not.

It is noteworthy that the *mtcu-1* or *mtcu-2* mutations promote ${\text{mt-tRNA}}_{\text{UAA}}^{\text{Leu}}$ and mt-tRNA^Gln^ accumulation since higher levels of these tRNAs were found in RNA purified from the mutant strains. These data suggest that *mtcu-1* and *mtcu-2* inactivation triggers a signaling pathway that promotes the overexpression or stabilization of mt-tRNAs, as the steady-state levels of these molecules ultimately depend on nDNA-encoded proteins. Previous studies with *E*. *coli* and yeast have shown that the overexpression of certain substrate tRNAs suppresses the phenotype of mutants lacking the U_34_ modification enzymes [69–71]. The accumulation of ${\text{mt-tRNA}}_{\text{UAA}}^{\text{Leu}}$ and mt-tRNA^Gln^ in the *mtcu-1* and *mtcu-2* mutants could, therefore, represent an attempt to compensate the effects of the mt-tRNA hypomodification in these strains. In contrast, we observed a decrease of the ${\text{mt-tRNA}}_{\text{UAA}}^{\text{Leu}}$ and mt-tRNA^Gln^ steady-state levels in the *mttu-1* single mutant. Considering that mt-tRNA^Gln^ is not a substrate for MTTU-1, the decrease in the levels of this mt-tRNA cannot be attributed to its modification status. Therefore, loss of *mttu-1* seems to activate a nuclear adaptive response that reduces the steady state levels of mt-tRNAs, in opposition to that observed in the *mtcu-1* and *mtcu-2* mutants.

The idea that retrograde signaling (and, therefore, the initial mitochondrial stress) is different in the two types of mutants is also supported by our results with genes encoding mitochondrial chaperons. The finding that the expression of the *hsp6*_*p*_::GFP reporter is up-regulated in the *mtcu-1* and *mtcu-2* mutants, but down-regulated in the *mttu-1* mutant is striking and provides evidence that loss of each type of U_34_ modifications generates a different mitochondrial stress. The role of modifications at position 2 and 5 of U_34_ may differ to some extent [72, 73], and their lack may, therefore, have different consequences. Notably, loss of U_34_ modifications in a subset of cytoplasmic tRNAs leads to ribosome pausing at their cognate codons in *S*. *cerevisiae* and *C*. *elegans*, which in turn results in the accumulation of misfolded proteins and proteotoxic stress [71]. Moreover, the U_34_ modifications introduced in *E*. *coli* tRNAs by the orthologues of MTTU-1, MTCU-1 and MTCU-2 are crucial for modulating the relative efficiency of anticodons in reading cognate codons [72, 73]. Therefore, one possible explanation for our results could be that the speed at which codons are read by mitochondrial ribosomes is differently affected by mutations in *mtcu-1*, *mtcu-2* or *mttu-1* and that loss of the different types of modification leads to pausing at different codons and, accordingly, to different spectra of misfolded proteins. Other mechanisms, not necessarily mutually exclusive, by which mutations *mtcu-1*, *mtcu-2* or *mttu-1* may regulate retrograde signaling are also plausible. For instance, the hypomodified mt-tRNAs could themselves act as signaling molecules [74], or the U_34_ modifying proteins, besides modifying mt-tRNAs, may have additional roles [17, 20].

It has been reported that stoichiometric imbalance between mtDNA- and nDNA-encoded OXPHOS subunits acts as an initial signal for a tissue-specific activation of stress responses, and that this signal results in a systemic change in metabolism independently of respiratory chain defects [75]. Our transcriptional analysis suggests that inactivation of the U_34_ modifying enzymes might trigger metabolic reprogramming including up-regulation of anaplerotic pathways (UCP4-dependent succinate transport and glutaminolysis), and down-regulation of glycolysis and fatty acid oxidation. Furthermore, the reprogramming differs depending upon the type of modification affected. Thus, the glyoxylate cycle appears to be activated in mutant *mttu-1* but not in the *mtcu-1* and *mtcu-2* single mutants. Thus, these data also support the proposal that the retrograde signaling triggered by the inactivation of MTTU-1 is different from that triggered by loss of MTCU-1 or MTCU-2.

Patients with point mutations in *TRMU* or *MTO1* or *GTPBP3* (homologues of *mttu-1*, *mtcu-2* and *mtcu-1*, respectively) show a reduction in the activity of the mtDNA-dependent respiratory complexes, especially of complexes I and IV. Our data indicate that the *C*. *elegans mttu-1*, *mtcu-1* and *mtcu-2* single mutants all display a mild OXPHOS dysfunction with impairment of complex I. Considering that the AMP/ATP ratio remained unchanged in the single mutants, a reasonable assumption is that some kind of compensatory mechanism maintains ATP levels. Glycolysis is likely down-regulated in all three single mutants since the mRNA levels of *pfk-1*.*1* and *ldh-1* were severely decreased. On the other hand, in all three mutants there was a significant induction of *ucp-4*. Human UCP4 has been shown to increase ATP synthesis by specifically interacting with complex II [76]. Moreover, *C*. *elegans* UCP-4 has been reported to regulate mitochondrial succinate import and complex II-metabolism [45]. Therefore, one possibility is that in the *C*. *elegans* single mutants, compensation for the partial impairment of complex I occurs through complex II activity sustained by UCP-4. This would explain the higher sensitivity of the mutants to the complex II inhibitor since activity of complex II would be especially required in a context where function of complex I is compromised. The reorganization of metabolism occurring in the *C*. *elegans* single mutants allows ATP and oxygen consumption levels to be maintained. However, the adaptive response has a biological cost as evidenced by the decreased fertility of the single mutants, especially in the case of the *mttu-1* animals, whose reproductive cycle is also affected. The relative fragility of this “survival mode” is also revealed when the single mutants grow under stress conditions (25°C) or when mutations affecting modification at position 2 and 5 are combined. Therefore, the animal models presented here support the idea that pathology may develop not as a direct consequence of a bioenergetic defect, but from the cell’s maladaptive response to the hypomodification status of mt-tRNAs.

Analysis of the expression of chaperone genes in the single mutants provides some interesting findings on the regulation of both genes. In worms, the UPR^mt^ signaling pathway is not completely understood, but several factors are known to be required. The induction of UPR^mt^ targets depends on the translocation to the nucleus of the ATFS-1 transcription factor as a result of the activity of a protease, CLPP. ATFS-1 acts together with two other proteins, UBL-5 and DVE-1, to activate chaperone expression [4, 40, 41]. Our data indicate that induction of the *hsp-6*_*p*_::GFP reporter in the *mtcu-1* and *mtcu-2* mutants does not require increased expression of *clpp-1* and *ubl-5* mRNA levels. In contrast, *clpp-1* mRNA levels are reduced in the *mttu-1* mutant. The reduced expression of *clpp-1* might explain why the *hsp-6*_*p*_::GFP reporter shows reduced expression in this mutant. However, it has been recently reported that in mammals the CLPP-1 homolog is not required for the UPR^mt^ [77].

The finding that in the *mtcu-1* and *mtcu-2* mutants the *hsp-6*_*p*_::GFP reporter is induced but the *hsp-60*_*p*_::GFP reporter is not may reflect a difference in the regulation of the *hsp-6* and *hsp-60* promoters or, alternatively, differential sensitivities of the two reporters to the same mitochondrial stress. Both possibilities have been previously proposed in relation to the differential response of the *hsp-6*_*p*_::GFP and *hsp-60*_*p*_::GFP reporters to other types of mitochondrial stress [78]. In this respect, it is worth noting that in mammalian cells, differential expression of proteins homologous to HSP-6 and HSP-60 has been observed under specific stress conditions [41].

It is notable that loss of both types of modification at U_34_, s^2^ and cmnm^5^, in *C*. *elegans* mt-tRNAs is associated with lethality, developmental arrest and germline defects. Lethality and mitochondrial dysfunction are features reminiscent of those found in *E*. *coli* and yeast where the combination of null mutations in the *mttu-1* and *mtcu-1* or *mtcu-2* orthologues confers synthetic lethality under certain growth conditions, as well as a respiratory defect in *S*. *cerevisiae* [17, 32, 33]. Our data suggest that in the *C*. *elegans* double mutant, the OXPHOS system is so severely impaired that it cannot be rescued by any adaptive mechanism. The dramatic biological effects on growth and survival are likely a consequence of drastic changes in the OXPHOS function and cell metabolism. Our study provides the first animal model reporting the effect of removing the orthologues of human TRMU and MTO1 (bacterial MnmA and MnmG) in the same individual.

The maternally rescued *mtcu-2; mttu-1* double mutant worms live almost twice as long as wild-type animals. Our data suggest that this phenotype is due to effects on several different longevity pathways. However, further work is needed to understand the mechanistic details underlying the extended lifespan of the *mtcu-2; mttu-1* double mutant.

In brief, we present here *C*. *elegans* models for the human diseases associated with hypomodification at position 2 and 5 of U_34_ in a mt-tRNA set, which exhibit different phenotypes: liver failure (TRMU) or hypertrophic cardiomyopathy (GTPBP3 and MTO1). Our results show that the inactivation of the *C*. *elegans* orthologues produces different nuclear expression patterns depending on whether the defect affects the modification at position 2 (MTTU-1) or 5 (MTCU-1 and MTCU-2) of U_34_. Our data suggest that the organism’s maladaptive response to defects in mt-tRNA modification should be taken into consideration in looking for specific therapeutic strategies for treating patients with mutations in *TRMU*, *GTPBP3* or *MTO1*. Finally, the combination of *mttu-1* and *mtcu-2* mutations in *C*. *elegans* establishes a new model to further investigate the relationships between the OXPHOS dysfunction and extended lifespan.

## Materials and methods

### Ethics statement

No vertebrate animals were used for these studies and no ethical approval was required.

### Nematode and *E*. *coli* strains

All nematode strains were derived from the wild-type Bristol strain N2 and were maintained as described [77]. Growth was at 20°C unless otherwise indicated. The following alleles and transgenic lines were obtained from the Caenorhabditis Genetics Center: *mtcu-2*(*ok2309*), *mtcu-1*(*ok3674*), *zcIs9*[*hsp-60*_*p*_::GFP] and *zcIs13*[*hsp-6*_*p*_::GFP]. The *mttu-1*(*tm3160*) and *mtcu-1*(*tm5041*) alleles where generated by Dr. Shohei Mitani with the Japanese Bioresource Project. At the beginning of this work, only one deletion mutant, *mtcu-1*(*ok3674*), appeared in the collection. However, our PCR analysis of the deletion indicated that it did not affect the *mtcu-1* gene. Later, the mutant *mtcu-1*(*tm5041*) was generated by the Japanese BioResource Project and then incorporated into this study as it was the only deletion mutant with the *mtcu-1* gene being affected. Knock-out mutations were confirmed by genotyping (primers used are listed in S2 Table) and all selected mutants were backcrossed seven to ten times with N2 wild-type before use. Double mutants were constructed by standard genetic crosses and animals with correct genotypes were selected by PCR (primers used are listed in S2 Table) or fluorescence signal/phenotypic traits. *nT1*[*qIs51*] was used as a green fluorescent balancer chromosome. The *E*. *coli* strains OP50 and HT115 were used to feed the worms under standard conditions and in RNAi experiments, respectively.

### Sequence analysis

The Blast homology searches were performed with the available web-based program BLASTP (Basic Local Alignment Search Tool Protein) of the National Center for Biotechnology Information (NCBI). Protein sequence alignments were carried out using seqweb program CLUSTAL OMEGA.

### APM-Northern blot analysis

Determination of the thiolation status of mt-tRNAs was carried out as previously described [18, 79]. Briefly, a volume of approximately 500 μl of worm pellet obtained from mixed-stage populations of liquid-cultured worms was processed using the “Isolation of small and large RNA” NucleoSpin miRNA kit (Macherey-Nagel) and the manufacturer’s instructions. 10 to 20 μg of total small RNA were run on 10% polyacrylamide/8 M urea gels with or without 0.01 mg/ml APM ([p-(N-acrylamino)-phenyl]mercuric chloride). APM was synthesized and kindly provided by Prof. Stephane Vincent [30]. Gels were run at 200 V for 3 h at 4°C and electroblotted onto positively charged nylon membranes. The RNA was crosslinked to the membrane by exposure to UV light for 2 min and baking at 80°C for 45 min. Pre-hybridization and hybridization were performed with Dig Easy Hyb (Roche) according to the manufacturer’s instructions. mt-tRNAs were detected with specific DIG-labeled synthetic oligodeoxynucleotides (S2 Table) used at 10 nM final concentration. The blots were detected using the DIG luminescent detection CDP-Star kit (Roche) according to the manufacturer´s instructions.

### In vitro angiogenin assay

Angiogenin nuclease-sensitivity assays were performed essentially as previously described [18] with minor modifications. In brief, 3 μg of total small RNA were mixed with 12.5 μg/ml of angiogenin (VITRO S.A.) in buffer (30 mM HEPES pH 7.4, 30 mM NaCl, 0.001% BSA). Mixtures were incubated at 37°C for different times (0, 0.5 h, 1 h, 1.5 h, 2 h or 3 h). Angiogenin was inactivated at 95°C for 10 min and subsequent freezing. The digested RNA samples were separated on 10% polyacrylamide, 8M urea gels and transferred to positively charged nylon membranes. The RNA was cross-linked to the membrane (60°C 1h) using freshly prepared EDC cross-linking solution [80–82]. ${\text{mt-tRNA}}_{\text{UAA}}^{\text{Leu}}$ and cyt-tRNA^Lys^ were detected with specific DIG-labeled oligodeoxynucleotides (S2 Table).

### mt-tRNA quantitation

Northern blotts were scanned and the images densitometrically analyzed using the ImageQuant software. Quantitative data were expressed as mean ± SD values and the signal intensity values of mt-tRNAs from the wild-type and mutant strains were compared using Student’s unpaired T-test.

### Mitochondrial DNA quantification

Mitochondrial DNA was quantified using Real Time-qPCR (Step One Real-Time PCR System, Applied Biosystems). The primers used to amplify *ama-1* nuclear gene and *ctb-1* mitochondrial gene are shown in S2 Table. Four synchronized L4 worms for each strain were lysed in a standard buffer (50 mM KCl, 10 mM Tris pH 8.2, 2.5 mM MgCl2, 0.45% NP-40 and 0.45% Tween 20) containing proteinase K for 1 h at 65°C. qPCR was performed using FastStart Universal SYBR Green Master PCR Mix (Roche). Data from at least three independent biological replicates were normalized to nuclear DNA and graphically represented in relation to the N2 wild-type strain.

### Western blot and protein quantification

For each sample, 100 synchronized young adult hermaphrodite worms were boiled for 10 min in 2X sample buffer (62.5 mM Tris pH 6.8, 10% glycerol, 10% SDS, 5% ß-mercaptoetanol and 0.002% bromophenol blue). Proteins were separated by gel electrophoresis and transferred to PVDF membranes. Blots were probed with anti-NDUFS3 (MS112, MitoSciences, 1:1000), anti-MT-CO1 (MS404, MitoSciences,1:1000) and anti-ATP synthase (provided by Erwin Knecht, 1:500) to analyze mitochondrial complex I NUO-2 (nuclear encoded), complex IV CTC-1(COX-1) (mitochondrial encoded) and complex V ATP-2 (nuclear encoded) expression, respectively. Actin was detected with abcam ab8227 (1:2000) antibody. Protein levels were measured by densitometry and normalized against actin.

### Mitochondrial membrane potential determination

TMRE (tetramethylrhodamine ethyl ester, Sigma, 87917-25MG), a fluorescent indicator of mitochondrial membrane potential as the uptake of this lipophilic cation depends on it [83, 84], was dissolved in DMSO at 50 μM (stock concentration) and applied to 10 ml NGM agar plates with OP50 bacteria to a final concentration of 0.1 μM. MitoTracker Red CMXRos (M-7512, Molecular Probes) was dissolved in DMSO at 1.25 mM (stock concentration) and applied to 10 ml MYOB agar plates with OP50 bacteria to final a concentration of 1.25 μM. Once the plates had dried, 10 larval stage 4 (L4) hermaphrodites of each strain were raised on the bacterial lawns in the dark for 16 h at 20°C. The animals were subsequently placed on fresh OP50 bacterial lawns for one additional hour in order to wash away excess fluorophore. For viewing, the same protocol as that used in the mitochondrial UPR induction experiment was followed.

### ATP measurement in *C*. *elegans* by HPLC

Perchloric acid extracts of worms were obtained as follows. 100 synchronized L4 hermaphrodite worms were washed twice with M9 buffer and left in about 70 μl of M9. 140 μl of 8% perchloric acid (v/v) (ice cold) were added to the pellet and worms were sonicated in a cold environment with five intermittent 30 s bursts. After sonication, 90 μl ice-cold 2.5 M KHCO_3_ were added to neutralize the perchloric acid. The samples were kept on ice for 5 min to cool. The supernatants were separated from the pellets by centrifugation at 12000 rpm for 20 min at 4°C. The supernatants were filtered and analyzed by HPLC as reported by Stocchi et al. [85], with slight modifications. The chromatographic conditions were the following: 9 min at 100% of Buffer A (0.1 M KH_2_PO_4_, pH 6), 6 min at up to 25% of Buffer B (Buffer A containing 10% (v/v) of CH_3_OH), 2.5 min at up to 90% of Buffer B, 2.5 min at up to 100% of Buffer B, and hold for 6 min. The gradient was then returned to the 100% of Buffer A in 2 min and maintained for 12 min to equilibrate the column. The flow rate was 1 ml/min. The column was Synergi fusion 5 μm. HPLC standards for ATP and AMP were used. Experiments were performed at least in triplicate.

### Rotenone and TTFA inhibition

Newly hatched L1 larvae were incubated on seeded NGM agar plates that contained different concentrations of rotenone (0, 0.001, 0.005, 0.01, 0.025, or 0.05 mM) or TTFA (0, 0.5, 0.75, 1, 2, or 3 mM) inhibitors. Four days later, survival was determined by counting the number of surviving larvae and the percentage of larvae at each stage of development. Experiments were performed in triplicate.

### Oxygen consumption measurements

Oxygen consumption rates were measured following published protocols on a Seahorse XF^e^96 respirometer [86, 87]. One-day old adult hermaphrodites of uniform size were used of wild-type or mutant strains that had been grown under standard conditions at either 20°C or 25°C. The parameters used were 2 min mixing, 1.5 min waiting and 3 min measurement. Each well contained approximately 10 worms and oxygen consumption rates of at least five separate wells were determined for each genotype. For measurements of the basal consumption rates, eight separate measurements were made for each sample and the average of all eight readings taken to be the rate for that sample. For the measurements of the maximal oxygen consumption rates in the presence of carbonyl cyanide 4-(trifluoromethoxy)phenylhydrazone (FCCP), eight measurements of the oxygen consumption rate were made but (following [86, 87]) since there is apparently a slight delay in FCCP entering worm mitochondria, the first two readings were not used in the calculations of the average maximal rates. Spare respiratory capacity (SPR) was calculated by subtracting basal OCR from maximal OCR.

### Mitochondrial UPR induction experiments

UPR^mt^ induction was achieved silencing L1 worms with *cyc-1*(RNAi) or treating L4 worms for 24 h with 1 mM of paraquat. For viewing, transgenic (induced or not) worms were mounted on agarose pads in the presence of 0.5 mM levamisole and examined in a Leica DM microscope equipped with both Nomarski differential interference contrast and epifluorescence optics. Images were captured with a Deltpix CCD camera and software (Deltapix, Copenhagen). All the images were taken at the same magnification, 10x, and with the same camera settings. To quantify the fluorescence, the images were transformed to greyscale and analyzed in ImageJ.

### RNA isolation and qRT-PCR

Total RNA was extracted from a synchronized L4 population of worms using Trizol reagent (Invitrogen). Total RNA was quantified spectrometrically (NanoDrop) before proceeding to subsequent steps. To quantify mRNA levels, one-step qRT-PCR was performed in an Applied Biosystems Step-One Real-Time PCR System. 50 ng of total RNA were reverse transcribed and amplified by qPCR in 20 μl of total volume reaction containing specific primers (Sigma) (S2 Table), Power SYBR Green PCR Mastex Mix, MultiScribe Reverse Transcriptase, and RNase Inhibitor (all from Applied Biosystems), according to the manufacturer’s instructions. *act-1* was used as an endogenous control.

### Determination of the Self-Brood size and length of reproductive cycle

Determination of the Self-Brood size was performed using ten L4 synchronized hermaphrodites and following standard procedures [88]. For assessing the length of the reproductive cycle, five young adult hermaphrodites (one-day adult) were manually picked and transferred to a new plate for 1 h. Young adult hermaphrodites were taken away and that time was established as t = 0. Plates were incubated at the experimental temperature. After two days, L3-L4 worms were transferred individually to new plates and they were monitored for the time they laid the first eggs.

### RNAi by feeding assay

The RNAi clones used here were obtained from the RNAi feeding library constructed by Julie Ahringer’s group at The Wellcome CRC Institute (University of Cambridge, Cambridge, UK) and distributed by Source BioScience, with the exception of *mttu-1*(RNAi) which was constructed in this work (see S1 Text). Plasmids were transformed into HT115 *E*. *coli* competent cells as indicated. *let-92*(RNAi) and the empty plasmid L4440 were transformed into HT115 *E*. *coli* cells to be used as a positive and negative controls, respectively. For the RNAi treatment, worms were synchronized by hypochlorous acid treatment and L4 animals were transferred to the NGM plates with HT115 bacteria expressing the dsRNA. Lifespan assays were performed directly on those L4 worms, while for the remaining RNAi assays the phenotypes of the progeny of the silenced L4 worms were assessed (unless otherwise specified). In order to analyze the number of progeny, the silenced one-day adult worms were transferred to a new plate containing bacteria expressing the dsRNA. When nematodes had finished laying eggs, they were removed from the plate. Worms were imaged on plates with a Nikon Digital Sight DS-L1 camera.

### Gonad dissection and DAPI staining

One-day adult worms were picked onto a fresh plate containing no bacteria. After some minutes, worms were picked again and immersed in 5 μl of PBS containing 0.25 mM levamisole on a microscope slide. Using a scalpel, worms were cut in the middle of the body, which resulted in gonad extrusion. Dissected gonads were fixed by adding 5 μl of 4% solution of paraformaldehyde in dilute sodium hydroxide for 1 h in a humidified chamber. After the incubation, most of the solution was dried and the dissected gonads were incubated with 5 μl of dimethylformamide (previously prechilled to -20°C). Fixed gonads were washed with PBS 1x + 0.1% Tween 20 and then 5 μl of ProLong Gold solution with DAPI (1 μg/ml) were added. Images were taken using LEICA DM 6000B microscope at 100x magnification in DIC and DAPI fluorescence.

### Lifespan assay

The lifespan assays were carried out at 20°C. Worms were transferred every day until they stopped laying eggs. For each strain, we randomly picked 25–30 L4 larvae worms from a synchronous population and transferred them to plates with OP50 or HT115-expressing dsRNA bacteria to start the lifespan experiment. Dead and alive worms were monitored every 1–2 days until death. Worms were scored as dead when they no longer responded to a mechanical stimulus. Worms that crawled off the plates during the assay were replaced from a backup group of the same age. Censoring occurred if animals desiccated on the edge of the plate or suffered from internal hatching.

### Statistical analysis

Data were expressed as individual data points, means ± SD. The statistically significant differences between WT and mutants were calculated with GraphPad Prism 6 software (GraphPad Software, Inc.) using Student’s unpaired t-test for P value, otherwise indicated. The statistically significant differences between means were indicated by asterisks [p < 0.05 (*), p < 0.01 (**) or p < 0.001 (***)].

## Supporting information

S1 Fig *C*. *elegans* proteins MTTU-1, MTCU-1 and MTCU-2 localize to mitochondria when expressed in yeast. (**A**) Schematic drawing of the distribution of exons and introns in the B0035.16 (*mttu-1*), F39B2.7 (*mtcu-1*) and F52H3.2 (*mtcu-2*) genes. Boxes and lines represent exons and introns, respectively. The red lines underneath denote the regions deleted in the alleles used in this study. The *mttu-1*(*tm3160*) deletion removes 592 bp of gene sequence spanning exons 5 and 6, and a part of intron 6. *mttu-1*(*tm3160*) lacks sequences encoding the region containing cysteine 205, which lies in the catalytic site; the corresponding residue of *E*. *coli* MnmA (C119) has been shown to be indispensable for the tRNA modification function [89]. *mtcu-2*(*ok2309*) is a deletion of 1340 bp spanning exons 2 to 5. The protein predicted to be encoded by *mtcu-2*(*ok2309*) lacks residues corresponding to those of *E*. *coli* MnmG involved in FAD or tRNA binding that have been shown to be crucial for activity [90–92]. For example, the deleted region includes C287, which corresponds to the catalytic C277 residue in *E*. *coli* MnmG numbering [92]. Finally, *mtcu-1*(*tm5041*) carries a 13 nucleotide insertion in place of a 597-bp deleted sequence spanning the 5´-UTR and exon 1. Notably, the deletion also affects the F39B2.5 gene, which belongs to the CEOP1760 operon and encodes for an orthologue of the SOCS6 and SOCS7 human proteins. The complex genetic alteration in MTCU-1 deletes the initiation codon and sequences encoding a part of the protein involved in binding of tetrahydrofolate, the donor of the methylene carbon that is directly attached to C5 of U_34_ through the tRNA modification reaction [93, 94]. (**B**) Micrographs of *S*. *cerevisiae* cells containing recombinant MTTU-1, MTCU-1 or MTCU-2 proteins fused at their C-termini to GFP (see S1 Text). Mitochondria were labeled with the fluorescent dye, MitoTracker. The merged signal (yellow) indicates co-localization of GFP (green) with the mitochondrial marker MitoTracker (red). Note that the GFP signal shows extensive overlap with that from MitoTracker indicating that the fusion proteins localize to *S*. *cerevisiae* mitochondria.
(TIF)

S2 Fig Antioxidant treatment does not reduce induction of the *hsp-6* promoter in the *mtcu-1* and *mtcu-2* single mutants. (**A-C**) 5 mM NAC treated and untreated *mtcu-1* and *mtcu-2* mutants expressing *hsp-6*_*p*_::GFP were examined at L4 larval stage. Representative images (A). Quantification is shown in (B) and (C). (**D-E**) The NAC treatment reduced the induction of the *hsp-6*_*p*_::GFP reporter mediated by paraquat (1 mM). Representative images (D). Quantification is shown in (E). **p<0.01, ***p<0.001. Statistical significance was evaluated with Student’s unpaired t-test. Error bars indicate standard deviation (SD).
(TIF)

S3 Fig RNAi of *mttu-1* in the *mtcu-2* mutant background causes slow growth and sterility. The right panels show time-lapse images of the progeny of *mtcu-2* hermaphrodites placed on bacteria expressing *mttu-1*(RNAi) from a plasmid. Those on the left show the progeny of *mtcu-2* hermaphrodites placed on a control bacterial strain containing the empty vector. *mtcu-2* hermaphrodites were placed on the plates as L4 larvae and were grown at 25°C. Note that the *mttu-1*(RNAi); *mtcu-2* larvae grew more slowly that control larvae and that they developed into sterile adults.
(TIF)

S4 Fig RNAi of *mtcu-2* in the *mttu-1* mutant background causes developmental arrest. The right panels show time-lapse images of the progeny of *mttu-1* hermaphrodites placed on bacteria expressing *mtcu-2*(RNAi). Those on the left show the progeny of *mttu-1* hermaphrodites placed on a control bacterial strain containing the empty vector. *mttu-1* hermaphrodites were placed on the plates as L4 larvae and were grown at 25°C. Note that the majority of the *mttu-1*; *mtcu-2*(RNAi) eggs failed to hatch. Larvae from eggs that did hatch failed to grow.
(TIF)

S1 Table The percentages give the amino acid identities and similarities (in parentheses) respectively. The predicted proteins MTTU-1, MTCU-1 and MTCU-2 contain 375, 439 and 638 amino acids and have molecular masses of ≈42.8, 48.7 kDa, and 71.7 kDa, respectively. They are very similar in size to their bacterial and eukaryotic orthologues. MitoProt II analysis of the sequences of the *C*. *elegans* proteins revealed mitochondrial targeting sequences for MTCU-1 at residue 41 and for MTCU-2 at residue 13 with high confidence (0.99 and 0.94, respectively). In contrast, no clear predicted mitochondrial targeting sequence was found for MTTU-1 in this analysis.
(PDF)

S2 Table Oligos used in this work. (PDF)

S1 Text Supplementary text extending material and methods. (PDF)
