# Supplementary figures and images for: Mutations in the Caenorhabditis elegans orthologs of human genes required for mitochondrial tRNA modification cause similar electron transport chain defects but different nuclear responses
Source: PLoS Genet. 2017 Jul 21;13(7):e1006921. doi: 10.1371/journal.pgen.1006921 (PMC5544249; doi:10.1371/journal.pgen.1006921)

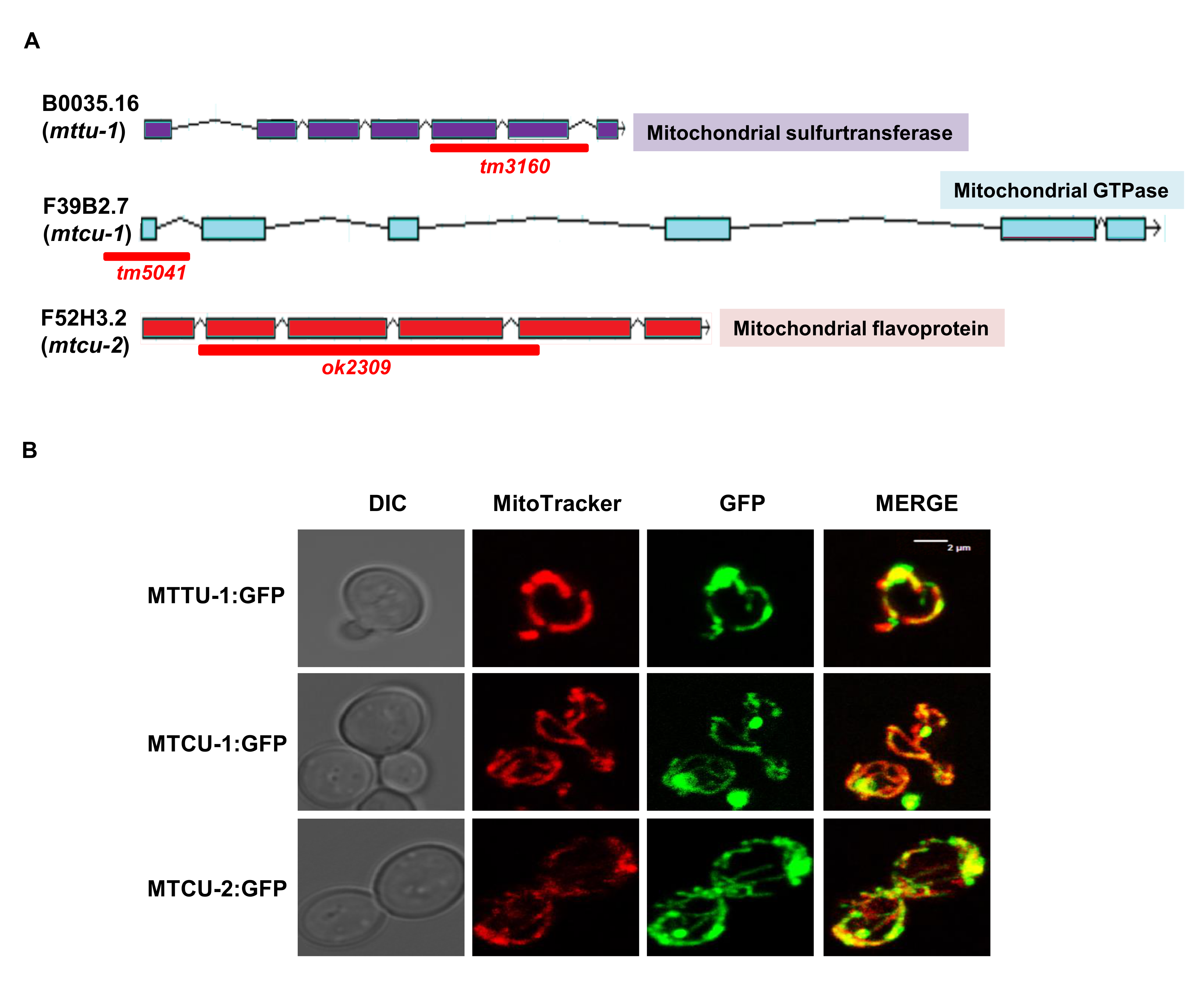

Supplement: S1 Fig — (A) Schematic drawing of the distribution of exons and introns in the B0035.16 (mttu-1), F39B2.7 (mtcu-1) and F52H3.2 (mtcu-2) genes. Boxes and lines represent exons and introns, respectively. The red lines underneath denote the regions deleted in the alleles used in this study. The mttu-1(tm3160) deletion removes 592 bp of gene sequence spanning exons 5 and 6, and a part of intron 6. mttu-1(tm3160) lacks sequences encoding the region containing cysteine 205, which lies in the catalytic site; the corresponding residue of E. coli MnmA (C119) has been shown to be indispensable for the tRNA modification function [89]. mtcu-2(ok2309) is a deletion of 1340 bp spanning exons 2 to 5. The protein predicted to be encoded by mtcu-2(ok2309) lacks residues corresponding to those of E. coli MnmG involved in FAD or tRNA binding that have been shown to be crucial for activity [90–92]. For example, the deleted region includes C287, which corresponds to the catalytic C277 residue in E. coli MnmG numbering [92]. Finally, mtcu-1(tm5041) carries a 13 nucleotide insertion in place of a 597-bp deleted sequence spanning the 5´-UTR and exon 1. Notably, the deletion also affects the F39B2.5 gene, which belongs to the CEOP1760 operon and encodes for an orthologue of the SOCS6 and SOCS7 human proteins. The complex genetic alteration in MTCU-1 deletes the initiation codon and sequences encoding a part of the protein involved in binding of tetrahydrofolate, the donor of the methylene carbon that is directly attached to C5 of U34 through the tRNA modification reaction [93, 94]. (B) Micrographs of S. cerevisiae cells containing recombinant MTTU-1, MTCU-1 or MTCU-2 proteins fused at their C-termini to GFP (see S1 Text). Mitochondria were labeled with the fluorescent dye, MitoTracker. The merged signal (yellow) indicates co-localization of GFP (green) with the mitochondrial marker MitoTracker (red). Note that the GFP signal shows extensive overlap with that from MitoTracker indicating that the fusi [file pgen.1006921.s001.tif]

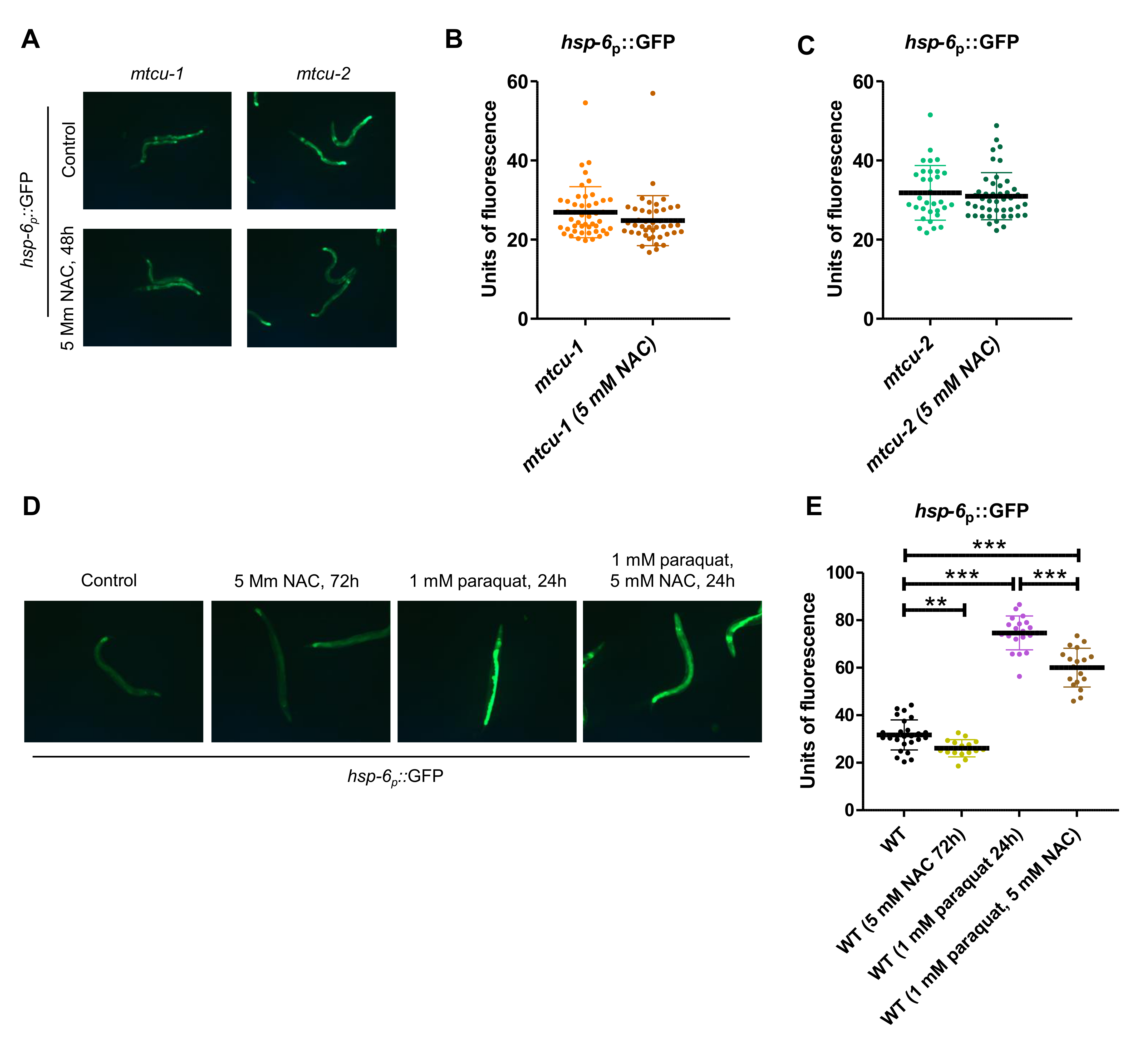

Supplement: S2 Fig — (A-C) 5 mM NAC treated and untreated mtcu-1 and mtcu-2 mutants expressing hsp-6p::GFP were examined at L4 larval stage. Representative images (A). Quantification is shown in (B) and (C). (D-E) The NAC treatment reduced the induction of the hsp-6p::GFP reporter mediated by paraquat (1 mM). Representative images (D). Quantification is shown in (E). **p<0.01, ***p<0.001. Statistical significance was evaluated with Student’s unpaired t-test. Error bars indicate standard deviation (SD). (TIF) [file pgen.1006921.s002.tif]

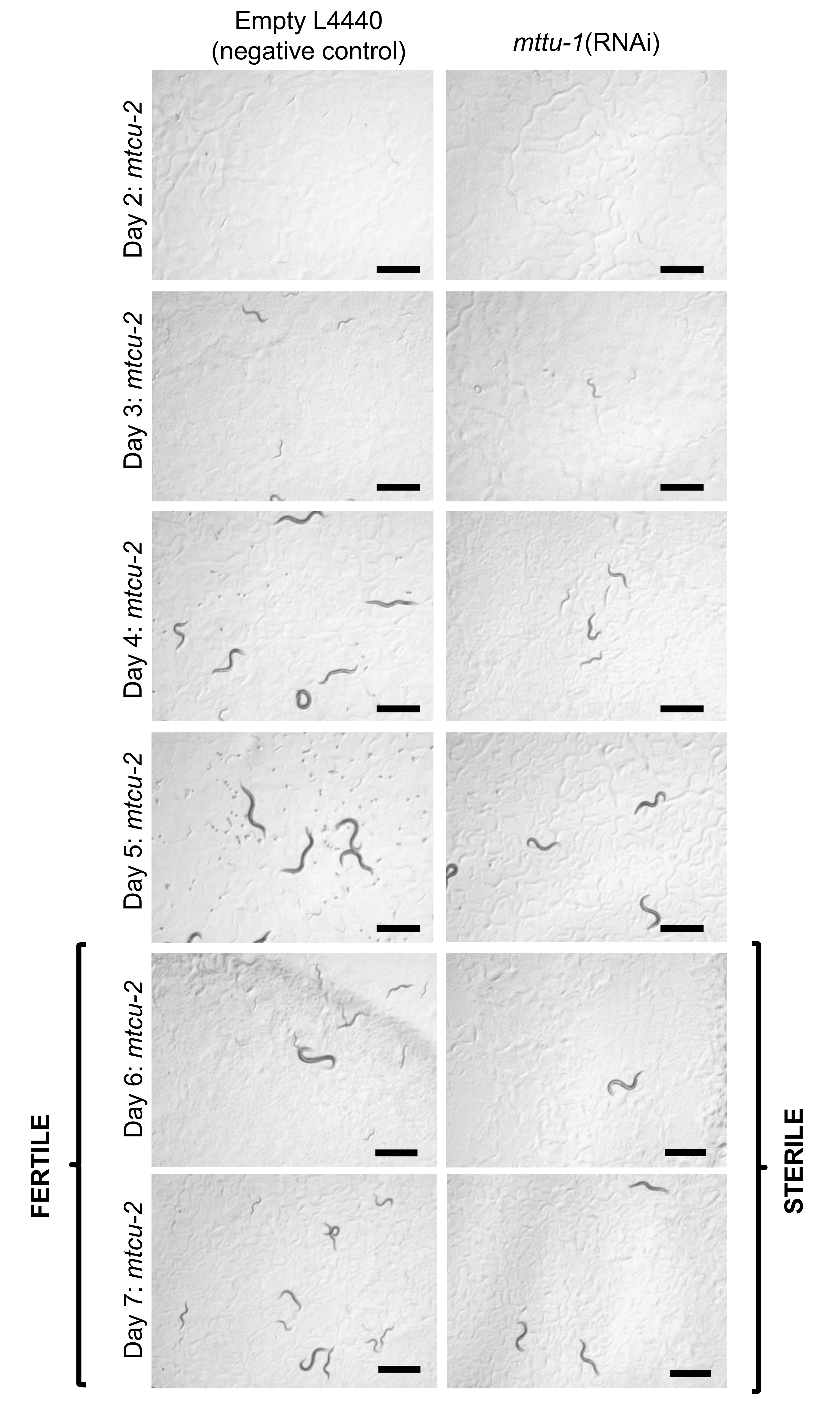

Supplement: S3 Fig — The right panels show time-lapse images of the progeny of mtcu-2 hermaphrodites placed on bacteria expressing mttu-1(RNAi) from a plasmid. Those on the left show the progeny of mtcu-2 hermaphrodites placed on a control bacterial strain containing the empty vector. mtcu-2 hermaphrodites were placed on the plates as L4 larvae and were grown at 25°C. Note that the mttu-1(RNAi); mtcu-2 larvae grew more slowly that control larvae and that they developed into sterile adults. (TIF) [file pgen.1006921.s003.tif]

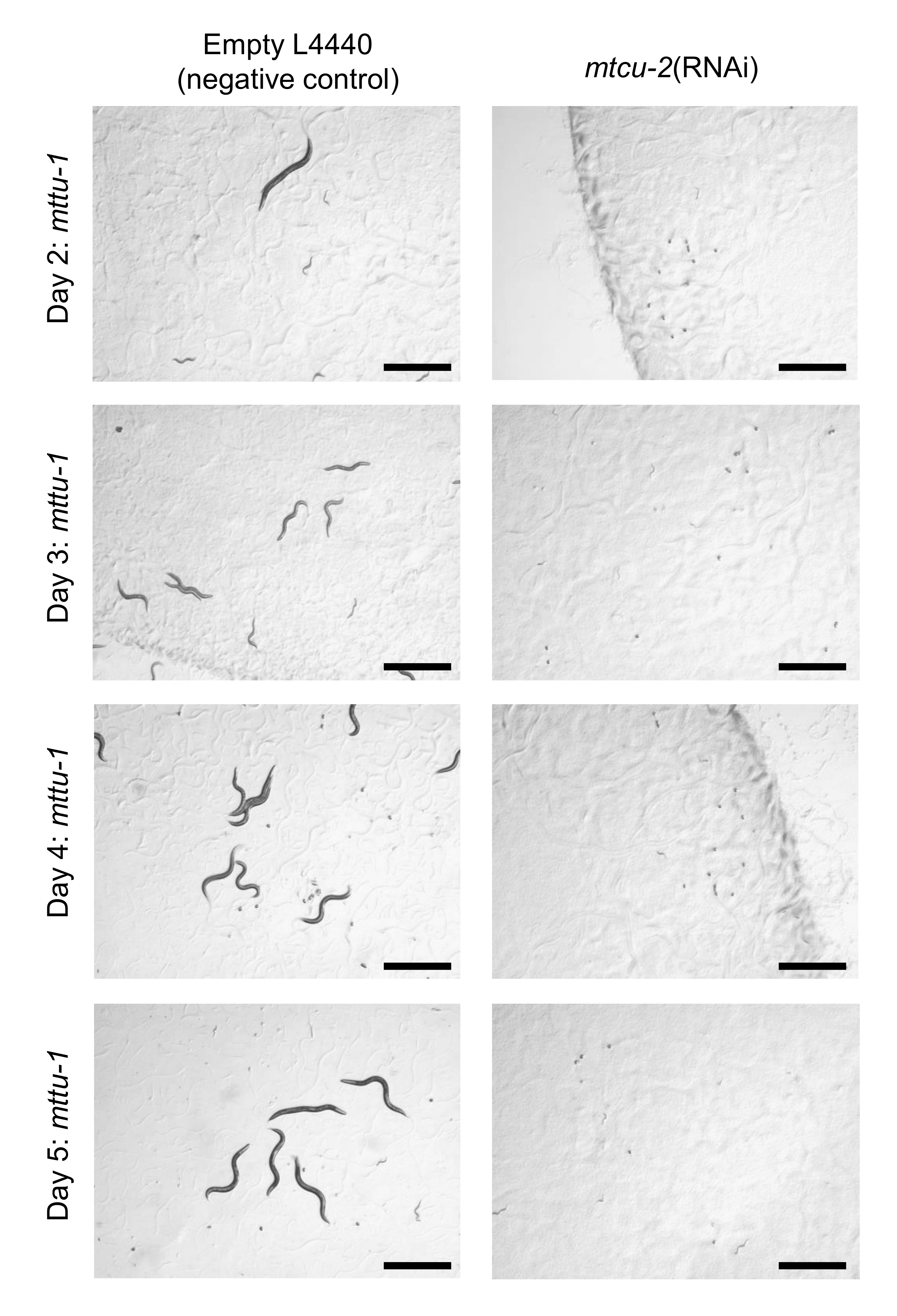

Supplement: S4 Fig — The right panels show time-lapse images of the progeny of mttu-1 hermaphrodites placed on bacteria expressing mtcu-2(RNAi). Those on the left show the progeny of mttu-1 hermaphrodites placed on a control bacterial strain containing the empty vector. mttu-1 hermaphrodites were placed on the plates as L4 larvae and were grown at 25°C. Note that the majority of the mttu-1; mtcu-2(RNAi) eggs failed to hatch. Larvae from eggs that did hatch failed to grow. (TIF) [file pgen.1006921.s004.tif]
