# Supplementary material for: Mutations in the Caenorhabditis elegans orthologs of human genes required for mitochondrial tRNA modification cause similar electron transport chain defects but different nuclear responses
Source: PLoS Genet. 2017 Jul 21;13(7):e1006921. doi: 10.1371/journal.pgen.1006921 (PMC5544249; doi:10.1371/journal.pgen.1006921)
Supplement: S1 Table — The predicted proteins MTTU-1, MTCU-1 and MTCU-2 contain 375, 439 and 638 amino acids and have molecular masses of ≈42.8, 48.7 kDa, and 71.7 kDa, respectively. They are very similar in size to their bacterial and eukaryotic orthologues. MitoProt II analysis of the sequences of the C. elegans proteins revealed mitochondrial targeting sequences for MTCU-1 at residue 41 and for MTCU-2 at residue 13 with high confidence (0.99 and 0.94, respectively). In contrast, no clear predicted mitochondrial targeting sequence was found for MTTU-1 in this analysis. (PDF) [file pgen.1006921.s005.pdf]

S1 Table. Comparisons of *C. elegans* MTTU-1, MTCU-1 and MTCU-2 with their orthologues in *E. coli, S. cerevisiae* and *H. sapiens*.

C. elegans	***E. coli***	***S. cerevisiae***	***H. sapiens***
	MnmA	MTU1/SLM3	MTU1/TRMU
**MTTU-1**	40% (58%)	30% (45%)	40% (54%)
	MnmE	MSS1	GTPBP3
**MTCU-1**	28% (48%)	32% (51%)	33% (47%)
	MnmG	MTO1	MTO1
**MTCU-2**	41% (61%)	42% (62%)	48% (63%)
