# Supplementary material for: Mutations in the Caenorhabditis elegans orthologs of human genes required for mitochondrial tRNA modification cause similar electron transport chain defects but different nuclear responses
Source: PLoS Genet. 2017 Jul 21;13(7):e1006921. doi: 10.1371/journal.pgen.1006921 (PMC5544249; doi:10.1371/journal.pgen.1006921)
Supplement: S2 Table — (PDF) [file pgen.1006921.s006.pdf]

S2 Table. Oligos used in this work			
**Name**	**Sequence**	**Tm**	**Use**
**Primers used in strain genotyping**			
**ceMTO1-L**	CTGCCAGATTTCTTGGTCGT	64.2ºC	*mtcu-2* gene amplification (750 pb in WT and 1 kb in mutant)
**ceMTO1-R**	ACGCTGTTGATTTTTGCCTT	63.5ºC	
**MTO1-int**	TCCCGTAGTGATTACAACAC	57.5ºC	
**ecMTU1-L**	TCTCTCCTCCACCAAGGCAT	80.4ºC	*mttu-1* gene amplification (1,3 kb in WT and 650 pb in mutant)
**ecMTU1-R**	GGGACATCACATTGTCCACG	71.6ºC	
**F39+351-R**	ACAATTTCGTGTATTTCATC	59.3ºC	*mtcu-1* gene amplification (1,3 kb in WT and 650 pb in mutant)
**F39-870-F**	AACAAATCAAACACAATACAACC	59.2ºC	
**ceMSS1-L**	GGCACTGGAAAAGAGACAAGA	63.3ºC	*mtcu-1* gene amplification to detect *ok3674* variation
**ceMSS1-R**	ACGTGCTCAAAAACGAATCC	63.9ºC	
**Primers used in *mttu-1*(RNAi) construction**			
**F-BamHImttu**	CGAGTTGGGATCCGAATGTCCGGCGGTGTGG	86.8ºC	*mttu-1*(RNAi) cloning in L4440
**R-XhoImttu-B**	ATAAAGTCTCGAGAATATTCATAATCTCTCCTCCACC	71.1ºC	
**Primers used in GFP recombinant proteins constructed by homologous recombination**			
**reB00-F**	GAATTCGATATCAAGCTTATCGATACCGTCGACAATGAAAATGCCACGAGTTG	84.2ºC	MTTU-1:GFP recombinant protein construction (underlined region recombines with pGREG600)
**reB00-R2**	AGTTCTTCTCCTTTACTCATTCTCGAGGTCGATAAAGTTTCTTGAATATTC	76.7ºC	
**reF52-F**	GAATTCGATATCAAGCTTATCGATACCGTCGACAATGTCGACAATCTTCGCCC	85.5ºC	MTCU-2:GFP recombinant protein construction (underlined region recombines with pGREG600)
**reF52-R**	AGTTCTTCTCCTTTACTCATTCTCGAGGTCGATTTTCCGATACAAAATTTGC	81ºC	
**reGTPBP3-F**	GAATTCGATATCAAGCTTATCGATACCGTCGACAATGTGGCGGGGGCTTTGGAC	88.3ºC	MTCU-1:GFP recombinant protein construction (underlined region recombines with pGREG600)
**reGTPBP3-R**	AGTTCTTCTCCTTTACTCATTCTCGAGGTCGACTTGCCCACACAGAAGTCC	84.3ºC	
**Primers used for Northern Blot**			
**DIG-Gln**	[DIG]GCAATCAAACTCTTTTGCACCAAAAACAA	71.3ºC	mt-tRNA^{Gln} detection
**DIG-Leu**	[DIG]AGTTGACGGATATCTTTTGCGCTTAAAACA	72ºC	mt-tRNA^{Leu} detection
**DIG-5S rRNACe**	[DIG]CCGTCTCCGATCCAAGTACTAA	64ºC	5s rRNA detection
**DIG-cyt-Lys**	[DIG]ATGCTCTACCGACTGAGCTAGCCGGGC	77ºC	cyt-tRNA^{Lys} detection
**Primers used for mtDNA/nDNA ratio**			
**MTCE.21-F**	GTTTATGCTGCTGTAGCGTG	61.1ºC	*ctb-1* (mitochondrial)
**MTCE.21-R**	CTGTTAAAGCAAGTGGACGAG	61.4ºC	
**F36A4.7-F**	TGGAACTCTGGAGTCACACC	62.9ºC	*ama-1* (nuclear)
**F36A4.7-R**	CATCCTCCTTCATTGAACGG	64.4ºC	
**Primers used for mRNA levels quantitation (qRT-PCR)**			
**act1_F**	GAAGGAAATCACCGCTCTTG	63.6ºC	*act-1* (normalyzer)
**act1_R**	TCCACATCTGTTGGAAGGTG	63.7ºC	
**F-cts-1b**	ACGGATTGGCTAACCAAGAG	62.9ºC	*cts-1* (citrate sinthase)
**R-cts-1b**	TACGGCATGTCCATATCCTG	63.3ºC	
**SOD3-F**	TGGTGGTGGACACATCAATC	64.5ºC	*sod-3* (antioxidant response)
**SOD3-R**	TGCAAGTTATCCAGGGAACC	63.8ºC	
**SOD1-F**	CTCATGGTGGACCAAAATCC	64.1ºC	*sod-1* (antioxidant response)
**SOD1-R**	ACAACCATAGATCGGCCAAC	63.7ºC	
**CTL2-F**	CGTATCCAAAACCCCAAGTG	64ºC	*ctl-2* (antioxidant response)
**CTL2-R**	CGAAATGAGCCATCTCATCC	64.5ºC	
**GST4-F**	TGCTCAATGTGCCTTACGAG	64ºC	*gst-4* (antioxidant response)
**GST4-R**	CCGAATTGTTCTCCATCGAC	64.4ºC	
**GCS1-F**	GTCTCATCGCTTGCTTCAAC	62.6ºC	*gcs-1* (antioxidant response)
**GCS1-R**	ACTTCCGGGAATGTGAATCC	64.9ºC	
**Fgt1_F**	AGACAAGTGGGCCAGCTACTCA	67.2ºC	*fgt-1* (glycolysis)
**Fgt1_R**	GGTCCGGTGGCAAAGGA	67.4ºC	
**Glna1_F**	AGCCAAGTGGACGGCTTTT	65.9ºC	*glna-1* (glutaminase)
**Glna1_R**	TGCGCCAGCATTGATTAGC	67.3ºC	
**Acs17_F**	GGAGACTATCACTGGAGAAGCTATG	63.2ºC	*acs-17* (fatty acid oxidation)
**Acs17_R**	GAACTGCTTCGTCTCCAAGAGTAG	64.4ºC	
**Ldh1_F**	GAGAGAAGACTGACAACGAACACTG	65.3ºC	*ldh-1* (glycolysis)
**Ldh1_R**	GAACGACTGGAAGAGAAAGGTAGAC	64.3ºC	
**Ucp4_F_a**	CGAACTTAAAGATAATTGGCTAACTCA	63.3ºC	*ucp-4* (succinate transport)
**Ucp4_R_a**	CGACATCTGATGGAAGTGATACAA	64.9ºC	
**Acdh12_F**	CCGATGTTTTCACTGTGTTTGC	66.4ºC	*acdh-12* (fatty acid oxidation)
**Acdh12_R**	CAAACGCTCTTTCGACAATGAAT	66ºC	
**Pfk1.1_F**	GCTCGACTTTATCCGTCAGC	63.8ºC	*pfk-1.1* (glycolysis)
**Pfk1.1_R**	CAGCGCTGTTCATACCTTGA	64ºC	
**Clpp1_F**	GTCATTCGTGCCGAAGAAAT	63.9ºC	*clpp-1* (UPR^{mt})
**Clpp1_R**	TTGATCCGTTGTGAGTCTCG	64ºC	
**Icl1_F**	ACTGCCTTGTCAGGATCCAC	64.2ºC	*icl-1* (glyoxilate)
**Icl1_R**	GAATTCGGTGTTGAGGTCGT	63.9ºC	
**F48E8.3_F**	CTTTTGGCAGACCTGCTTTC	63.8ºC	F48E8.3 (malate dismutation)
**F48E8.3_R**	GCAGACACTGGGAACACCTT	64.2ºC	
**UBL5-F**	CACAAACTGGAACACGATGG	64.1ºC	*ubl-5* (UPR^{mt})
**UBL5-R**	CCCTCGTGAATCTCGTAATCC	64.5ºC	
