# Supplementary material for: Mutations in the Caenorhabditis elegans orthologs of human genes required for mitochondrial tRNA modification cause similar electron transport chain defects but different nuclear responses
Source: PLoS Genet. 2017 Jul 21;13(7):e1006921. doi: 10.1371/journal.pgen.1006921 (PMC5544249; doi:10.1371/journal.pgen.1006921)
Supplement: S1 Text — (PDF) [file pgen.1006921.s007.pdf]

Construction of *mttu-1*(RNAi) silencer

The primers used to clone *mttu-1*(RNAi) from cDNA are listed in S2 Table. The amplified region was digested with BamHI using the restriction site in F-primer and an internal restriction site in exon 6, and was cloned into the L4440 plasmid. The sequence of the *mttu-1*(RNAi) clone encloses exons 1 to 6.

Construction and expression of fluorescent fusion proteins in *Saccharomyces cerevisiae*

Fluorescent fusion proteins were used to address the subcelular localization of *C. elegans* MTTU-1, MTCU-1 and MTCU-2 in *S. cerevisiae*. The whole ORFs (cDNAs) of these 3 proteins, including their putative mitochondrial targeting sequences, were cloned by recombination into the pGREG600 plasmid in-frame with the amino terminal sequence of the GFP coding region under the *GAL1* promoter as described [1]. All primers used are described in S2 Table. Yeast transformation was carried out by the lithium acetate method [2]. The yeast strain used was BY4741 [MATa; his3Δ1; leu2Δ0; met15Δ0; ura3Δ0], obtained from the EUROSCARF collection (Heidelberg, Germany) [3].

Exposure to oxidants or antioxidants

For experiments with N-Acetyl-L-cystein (NAC) (Sigma) alone, age-synchronized L1 worms from the stated strains were grown at 20ºC for 48 h on fresh NGM plates containing 5 mM NAC or on control plates. For experiments with oxidants, L4 wild-type worms expressing *hsp-6*$_p$*::*GFP promoter were grown on plates containing either 1 mM paraquat , or on those containing both 1 mM paraquat and 5 mM NAC for 24 h. For viewing, the worms were mounted on 2% agarose pad and anaesthetized with 0.5 mM levamisole and examined in a Leica DM microscope. Images were captured with a

Deltapix CCD camera and software (Deltapix, Copenhagen). All the images were taken at the same magnification, 10x, and with the same camera settings. To quantify the fluorescence, the images were transformed to greyscale and analyzed in ImageJ.

References

1. Jansen G, Wu C, Schade B, Thomas DY, Whiteway M. Drag&Drop cloning in yeast. Gene. 2005;344:43-51. Epub 2005/01/20. doi: S0378-1119(04)00642-0 [pii] 10.1016/j.gene.2004.10.016. PubMed PMID: 15656971.
2. Ito H, Fukuda Y, Murata K, Kimura A. Transformation of intact yeast cells treated with alkali cations. J Bacteriol. 1983;153(1):163-8. Epub 1983/01/01. PubMed PMID: 6336730; PubMed Central PMCID: PMC217353.
3. Brachmann CB, Davies A, Cost GJ, Caputo E, Li J, Hieter P, et al. Designer deletion strains derived from Saccharomyces cerevisiae S288C: a useful set of strains and plasmids for PCR-mediated gene disruption and other applications. Yeast. 1998;14:115-32.
